# Integration of Transcriptome and Metabolome Provides Unique Insights to Pathways Associated With Obese Breast Cancer Patients

**DOI:** 10.3389/fonc.2020.00804

**Published:** 2020-05-19

**Authors:** Mohammed A. Hassan, Kaltoom Al-Sakkaf, Mohammed Razeeth Shait Mohammed, Ashraf Dallol, Jaudah Al-Maghrabi, Alia Aldahlawi, Sawsan Ashoor, Mabrouka Maamra, Jiannis Ragoussis, Wei Wu, Mohammad Imran Khan, Abdulrahman L. Al-Malki, Hani Choudhry

**Affiliations:** ^1^Department of Biochemistry, Faculty of Science, King Abdulaziz University, Jeddah, Saudi Arabia; ^2^Department of Basic Medical Sciences, College of Medicine and Health Sciences, Hadhramout University, Mukalla, Yemen; ^3^Department of Medical Laboratory Technology, Faculty of Applied Medical Sciences, King Abdulaziz University, Jeddah, Saudi Arabia; ^4^Center of Excellence in Genomic Medicine Research, King Fahd Medical Research Center, King Abdulaziz University, Jeddah, Saudi Arabia; ^5^Department of Pathology, Faculty of Medicine, King Abdulaziz University, Jeddah, Saudi Arabia; ^6^Department of Biological Sciences, Faculty of Science, King Abdulaziz University, Jeddah, Saudi Arabia; ^7^Immunology Unit, King Fahd Medical Research Centre, King Abdulaziz University, Jeddah, Saudi Arabia; ^8^Department of Radiology, King Abdulaziz University Hospital, Jeddah, Saudi Arabia; ^9^Department of Oncology and Metabolism, School of Medicine, University of Sheffield, Sheffield, United Kingdom; ^10^Department of Human Genetics, McGill University Genome Centre, McGill University, Montreal, QC, Canada; ^11^Department of Medicine, University of California, San Francisco, San Francisco, CA, United States; ^12^Cancer and Mutagenesis Unit, King Fahd Medical Research Center, King Abdulaziz University, Jeddah, Saudi Arabia

**Keywords:** transcriptomics, metabolomics, obesity, breast cancer, integration metabolism, whole blood, OLFM4

## Abstract

Information regarding transcriptome and metabolome has significantly contributed to identifying potential therapeutic targets for the management of a variety of cancers. Obesity has profound effects on both cancer cell transcriptome and metabolome that can affect the outcome of cancer therapy. The information regarding the potential effects of obesity on breast cancer (BC) transcriptome, metabolome, and its integration to identify novel pathways related to disease progression are still elusive. We assessed the whole blood transcriptome and serum metabolome, as circulating metabolites, of obese BC patients compared them with non-obese BC patients. In these patients' samples, 186 significant differentially expressed genes (DEGs) were identified, comprising 156 upregulated and 30 downregulated. The expressions of these gene were confirmed by qRT-PCR. Furthermore, 96 deregulated metabolites were identified as untargeted metabolomics in the same group of patients. These detected DEGs and deregulated metabolites enriched in many cellular pathways. Further investigation, by integration analysis between transcriptomics and metabolomics data at the pathway levels, revealed seven unique enriched pathways in obese BC patients when compared with non-obese BC patients, which may provide resistance for BC cells to dodge the circulating immune cells in the blood. In conclusion, this study provides information on the unique pathways altered at transcriptome and metabolome levels in obese BC patients that could provide an important tool for researchers and contribute further to knowledge on the molecular interaction between obesity and BC. Further studies are needed to confirm this and to elucidate the exact underlying mechanism for the effects of obesity on the BC initiation or/and progression.

## Introduction

Breast cancer (BC), the most common malignant tumor type, was ranked top in incidence with high prevalence and mortality among females in Saudi Arabia as well as worldwide (1–3). BC is a molecularly heterogeneous, complex, and multifactorial disease with different biological and clinical characteristics (4). A number of BC-related etiological factors have been identified as hereditary, genetic factors, environmental, and lifestyle risk factors (5). Obesity poses a serious public health issue worldwide (6). In Saudi Arabia, the prevalence of obesity is 28.7% with a higher incidence among women (7). Obesity is one of the risk factors associated with the development of many types of cancer including BC. A number of studies, a few of them in Saudi Arabia, have reported an association between obesity and BC among postmenopausal women whereas the inverse relationship was reported among premenopausal women, however, this association remains unclear (8–13).

Obesity–BC molecular interaction could provide an important tool for researchers, as it may help to identify and discover new molecular fingerprints, as well as clarify molecular mechanisms involved in screening and develop therapeutic strategies for the management of BC. Notably, a few studies have focused on the molecular interaction between obesity and BC but this association still remains unclear. Recently, omics techniques, such as transcriptomics and metabolomics, have been widely used to improve understanding of the underlying biological mechanisms and biomarkers identification (14). Many studies utilized transcriptomics to investigate human diseases at the molecular level and identify the variation in transcriptomic profile in relation to diagnostic, treatment, or management and that may help to understand the mechanisms of disease initiation and progression (15–19). Moreover, many transcriptomic studies have been based on the analysis of the association between BC and obesity (18, 20). On the other hand, metabolomic investigations have been widely utilized in cancer metabolism and biomarkers identification to deduce the onset and progression of cancer (21, 22). Increasingly, studies now include measurements from multiple omics techniques rather than the single omics technology of a set of samples in early studies (14). There is currently very limited published research that has investigated the integration between deregulated transcriptomics and metabolomics data profiling in BC blood liquid biopsy. Therefore, in our study, we performed the integration between deregulated transcriptomics data and metabolomics profiling in BC patients with obesity to provide a better understanding of the biological status and shed new insights into potential molecular mechanisms, the interactions and biomarkers in the relationship between obesity and BC. Pathways and network connections were carried out to further explore the relationship between the selected metabolites and candidate transcripts. This could give considerable importance for the clinical management of BC patients and could provide an important tool for researchers as well as to increasing the knowledge on the molecular interaction between obesity and BC.

## Materials and Methods

### Study Subjects

**Figure 1:**
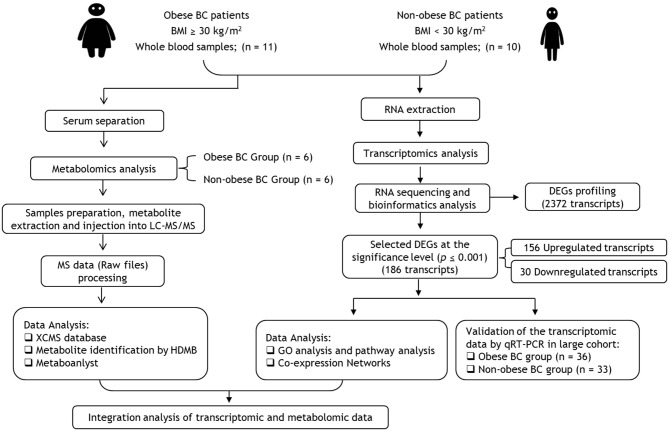
Flowchart of transcriptomics and metabolomics analysis in obese vs. non-obese BC patients. BC, Breast cancer; BMI, Body mass index; DEGs, differentially expressed genes; GO, Gene ontology.

We summarized the study workflow for identification and validation of signature RNAs and metabolites in obese BC patients in Figure 1. The study includes 69 newly diagnosed and before any treatment BC female subject who attended the Unit of Mammogram, Department of Radiography at King Abdulaziz University Hospital (KAUH), Jeddah, Saudi Arabia. The Unit of Biomedical Ethics, Research Committee, approved this study (Document number: HA-02-J-008). All BC patients signed the consent form. The patients' information was obtained through a standard questionnaire and the anthropometric data were collected using standard and well-established methods. The clinicopathological characteristics were obtained in collaboration with the Pathology Department at KAUH, Jeddah, Saudi Arabia.

The WHO recommendations (23) were used to classify BC patients as obese [Body mass index (BMI) ≥ 30 kg/m^2^, *n* = 36] and non-obese, which include lean and overweight (BMI < 30 kg/m^2^, *n* = 33). From all patients' cohort, RNA sequencing (RNA-seq) was performed for only 10 non-obese with lowest BMI and 11 obese BC patients with highest BMI. Then the transcriptomics data were validated in all groups, non-obese (*n* = 33) and obese (*n* = 36) BC patients. However, from the RNA samples cohort, only six samples of each group were selected for the metabolomics. These selections were based on BMI differentiation as with the RNA-seq samples selection.

### Blood and Serum Sample Collection and Storage

Whole blood samples were collected in PAXgene™ blood RNA tubes (PreAnalytiX, Switzerland) as well as in BD Vacutainer™ venous blood collection tubes: SST™ serum separation tubes (Fisher Scientific, USA), according to the manufacturer's instructions. Serum samples were aliquots after separated from the clotted blood. Blood samples in PAXgene™ blood RNA tubes, as well as aliquots serum samples, were stored at −80°C until used for transcriptome and metabolome analysis, respectively.

### Transcriptome Analysis

#### RNA Extraction

Total RNA was isolated from whole blood using the PAXgene^TM^ blood RNA kit (Qiagen, UK). The concentration and purity of the extracted RNA were verified by DeNovix DS-11 Spectrophotometer (DeNovix, USA) and Agilent 2100 bioanalyzer measurements (Agilent Technologies, USA). The RNA samples were stored at −80°C until used.

#### RNA Library Preparation, Sequencing, and Differentially Expressed Genes Analysis

The next-generation sequencing technologies were used for performed RNA-seq experiment to discover the amount of RNA in a blood biological sample at a given moment by using the Next-Seq 500 system (Illumina, Singapore) as described elsewhere (24). Approximately 2 μg of total RNA was fragmented and end-repaired using the Illumina directional protocol. Complementary DNA (cDNA) sequencing libraries were constructed using Illumina ®TruSeqTM stranded total RNA sample preparation kit (Illumina, USA) according to the manufacturer's instructions. The concentration and purity of the cDNA libraries were measured (RNA integrity number score > 7.0) using Agilent 2100 bioanalyzer (Agilent Technologies, USA). The libraries were sequenced using the Next-Seq 500 platforms in single-end 150-bp mode (Illumina, Singapore) according to the manufacturer's protocol. The FASTX-Toolkit (25) was used to remove adaptor sequences and to filtered low-quality base call and low-quality reads. TopHat2 program (26) was used to mapping short filtered sequencing reads to the human genome (UCSC) in order to identify exon-exon splice junctions, and the quantified genes expression level was done using Subreads package feature counts (27). They were tested after calculating the library size and appropriate data set dispersion depending on gene expression values by using edgeR Bioconductor package (28). Differentially expressed genes (DEGs) were presented as log fold change (logFC) and *p* ≤ 0.05 was counted statistically significant.

#### Gene Ontology and Pathway Analysis of Transcriptomic Data

Gene ontology (GO) analysis (29) was carried out to determine the functions of the DEGs identified. The highly significantly DEGs (*p* ≤ 0.001) were uploaded into Enrichr tool (30) for analysis and organized into groups basis of cellular components, biological processes, and molecular functions. The pathway enrichment analysis was also conducted for the highly significantly DEGs to place these target genes in the pathways according to the Kyoto encyclopedia of genes and genomes database (KEGG) database (31) by used Enrichr tool.

#### RNAs Co-expression and Interaction Network

The co-expression network of the most highly significant DEGs (*p* ≤ 0.0001) was constructed to identify the potential DEGs interaction by using GeneMANIA tool (32). A co-expression network was constructed according to the correlation analysis between the DEGs associated with obesity and BC.

#### Validation of the Transcriptomic Data by Quantitative Real-Time PCR

Total RNA (800 ng) was reverse transcribed using QuantiTect reverse transcription kit (Qiagen, UK). The expression of a selected gene(s) was measured in duplicate in a large cohort of BC blood patients (33 non-obese and 36 obese BC patients) by quantitative real-time PCR (qRT-PCR) using IQ SYBR green mix (Bio-Rad, USA) and RPL11 as the internal control on CFX Connect™ real-time PCR detection system (Bio-Rad, USA). The primers of selected genes were designed over two different exons and the sequences are available upon request. No-reverse transcriptase controls (NRCs) and no-template controls (NTCs) were included for each primer pair. The relative expression quantification was calculated depending on the 2^−ΔΔct^ methods (33).

### Metabolome Analysis

#### Samples Preparation and Metabolite Extraction

Among the 21 BC patients that subjected in RNA-seq assay; six obese BC and equal numbers of non-obese BC patients were selected for metabolomics. Serum metabolites were extracted and analyzed in triplicates, by liquid chromatography-tandem mass spectrometry (LC-MS/MS), for untargeted metabolomics detection. One-hundred microliters of serum used for metabolites extraction by ice-cold Methanol: Acetonitrile: water in a ratio of (2:1:1 volume/volume), that was added to serum and mixture was vortexed, followed by incubation at −20°C for an hour, samples were then spun at 4°C for 5 min at 8,000 rpm. Supernatants analyzed by LC-MS/MS. Each sample (10 μL) was injected individually into Hypersail gold high-performance liquid chromatography (HPLC) column (150 × 4.6 mm, 5 μm) with a flow rate of 0.250 ml/min and mobile phase A 0.1% formic acid in 99.9% acetonitrile formic acid (0.1%, volume/volume) and mobile phase B is 0.1% formic acid in MilliQ. Mass Spec parameter performed as an earlier report (34). Raw data processed using the online XCMS database (35). Isotopic peaks were integrated using the CAMERA (36). Metabolites were searched using the METLIN database (37) and the pathway analysis done with the help of MetaboAnalyst 3.0 (38).

### Statistical Analysis

Statistical analyses using unpaired, two-tailed *t*-tests were performed in GraphPad software Prism version 8.0.1 (GraphPad Software, La Jolla California USA, www.graphpad.com). The data were presented as a mean ± standard error of the mean (SEM). The level of significance was given at *p* ≤ 0.05.

## Results

### Transcripts Profiling Changes Between Obese and Non-obese BC Patients

The RNA-seq study included 21 female patients newly diagnosed with BC and before they underwent any treatment. The non-obese and obese BC patients were significantly different in the BMI, waist and hip circumference (Table 1). Conversely, non-obese and obese BC patients did not show any significant differences with general and clinicopathological characteristics (Supplementary Tables S1, S2). The extracted RNA from whole blood subjected for RNA-seq assay. The RNA-seq data detected a total of 31,698 RNA transcripts; among them, a total of 2,372 transcripts were found significantly dysregulated in obese BC patients compared with non-obese BC patients, of which 1,737 upregulated and 635 downregulated transcripts (Table 2). Moreover, 186 DEGs at the highly significance level (*p* ≤ 0.001) were identified, comprising 156 upregulated and 30 downregulated transcripts (Supplementary Table S3), furthermore, 31 DEGs were found as the most highly significantly (*p* ≤ 0.0001), of which 23 upregulated and 8 downregulated transcripts (Table 3). Among all identified DEGs; placenta growth factor (PGF) was the most downregulated gene with a logFC of −5.52, while adenylate cyclase type 1 (ADCY1) was the most upregulated gene with a logFC of 5.52 in obese BC patients compared with non-obese BC patients.

**Table 1:** Baseline characteristics of studied BC patients in RNA-seq analysis.

**Parameters**	**Non-obese BC**	**Obese BC**	***p*-value**
*N* (%)	10 (47.62)	11 (52.38)	
Age (years)	47.70 ± 2.16	49.09 ± 2.74	0.69
BMI (kg/m^2^)	22.10 ± 0.88	36.82 ± 1.87	<0.0001
Waist circumference (cm)	76.30 ±5.54	99.82 ± 3.61	0.0018
Hip circumference (cm)	90.20 ± 6.49	119.2 ± 3.92	0.0010

*Data were presented as mean ± SEM. BMI, body mass index; N, number of samples*.

**Table 2:** The total number of transcripts altered in obese BC compared with non-obese BC patients, the classification based on *p*-values.

	**All transcripts**		**Upregulated transcripts**		**Downregulated transcripts**		**Non-change transcripts**
***p*-value range**	**Transcripts number**	**LogFC range**	**Transcripts number**	**LogFC range**	**Transcripts number**	**LogFC range**	**Transcripts number**
All	31,698	−5.53 to 6.98	19,105	0.01 to 6.98	12,341	−5.53 to −0.01	252
≤0.05	2,372	−5.53 to 6.98	1,737	0.30 to 6.98	635	−5.53 to −0.26	–
≤0.01	851	−5.53 to 6.98	664	0.41 to 6.98	187	−5.53 to −0.42	–
≤0.001	186	−5.53 to 6.98	156	0.62 to 6.98	30	−5.53 to −0.73	–
≤0.0001	31	−5.53 to 5.52	23	2.65 to 5.52	8	−5.53 to −0.95	–

*LogFC rounded to two numbers after the decimal point. FC, fold change*.

**Table 3:** The most highly significantly DEGs in obese BC as compared with non-obese BC patients, ordered depending on LogFC.

	**Gene symbol**	**Gene name**	**Gene type**	**LogFC**	***p*-value**
1	ADCY1	Adenylate cyclase type 1	Coding	5.52	0.0001
2	ASPM	Abnormal spindle-like microcephaly-associated protein	Coding	4.33	<0.0001
3	E2F7	Transcription factor E2F7	Coding	4.18	0.0001
4	GALNT9	Polypeptide N-acetylgalactosaminyltransferase 9	Coding	4.10	0.0001
5	MYH10	Myosin-10	Coding	4.03	<0.0001
6	SEPT3	Neuronal-specific septin-3	Coding	3.90	0.0001
7	CDK1	Cyclin-dependent kinase 1	Coding	3.84	0.0001
8	AP001429.1	LncRNA-AP001429.1	Non-coding	3.74	<0.0001
9	IGFBP2	Insulin-like growth factor-binding protein 2	Coding	3.68	0.0001
10	BUB1B	Mitotic checkpoint serine/threonine-protein kinase BUB1 beta	Coding	3.50	<0.0001
11	CENPF	Centromere protein F	Coding	3.49	<0.0001
12	OLFM4	Olfactomedin-4	Coding	3.47	<0.0001
13	TOP2A	DNA topoisomerase 2-alpha	Coding	3.44	<0.0001
14	TICRR	Treslin	Coding	3.42	0.0001
15	CEP55	Centrosomal protein of 55 kDa	Coding	3.17	0.0001
16	UHRF1	ubiquitin like with PHD and ring finger domains 1	Coding	3.17	0.0001
17	SCN8A	Sodium channel protein type 8 subunit alpha	Coding	3.08	0.0001
18	SLCO4A1	Solute carrier organic anion transporter family member 4A1	Coding	3.02	<0.0001
19	CD109	CD109 antigen	Coding	2.97	0.0001
20	BRCA2	Breast cancer type 2 susceptibility protein	Coding	2.81	0.0001
21	MYB	Transcriptional activator Myb	Coding	2.74	0.0001
22	MKI67	Proliferation marker protein Ki-67	Coding	2.66	0.0001
23	ARHGEF10	Rho guanine nucleotide exchange factor 10	Coding	2.65	<0.0001
24	TIGD3	Tigger transposable element-derived protein 3	Coding	−0.95	0.0001
25	TPST1	Tyrosylprotein sulfotransferase 1,-like	Coding	−1.32	<0.0001
26	VSIG4	V-set and immunoglobulin domain-containing protein 4	Coding	−1.66	<0.0001
27	RNY1	RNA, Ro-Associated Y1	Non-coding	−2.24	0.0001
28	IGLV1-47	Immunoglobulin lambda variable 1-47	Coding	−2.75	<0.0001
29	IGKV1D-16	Immunoglobulin kappa variable 1D-16	Coding	−2.77	0.0001
30	IGHV6-1	Immunoglobulin heavy variable 6-1	Coding	−3.62	<0.0001
31	PGF	Placenta growth factor	Coding	−5.53	<0.0001

*LogFC rounded to two numbers after the decimal point. FC, Fold change*.

### Gene Ontology Enrichment, Pathway Analysis and RNA Co-expression Network of Circulating Transcriptomic Data

**Figure 2:**
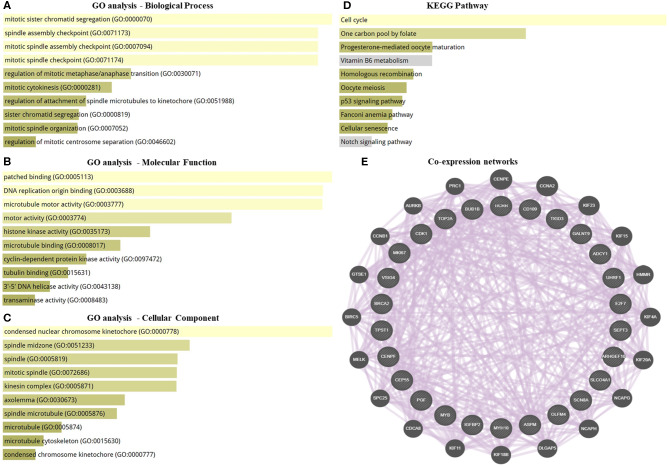
GO enrichment, pathway analysis, and co-expression network of the transcriptomic data. **(A–C)** GO analysis of DEGs that associated with biological process, molecular function, and cellular component. **(D)** KEGG pathway analysis for DEGs. **(E)** Co-expression networks of DEGs. Dysregulated genes interacted with 46 total genes by 2,863 total links. The association sorted by combined score ranking. DEGs, differentially expressed RNAs; GO, gene ontology; KEGG, Kyoto encyclopedia of genes and genomes database.

GO enrichment and KEGG pathway analysis of the highly significantly DEGs were performed to identify the gene product enrichment in various GOs categories and determine the DEGs functions. Deregulated genes were enriched in 234, 1,058, 126 targets in the GO molecular function, GO biological process and GO cellular component, respectively. As shown in Figures 2A–C, the highest enriched GO's targeted were associated with the mitotic sister chromatid segregation (GO:0000070) in the GO biological process analysis. Meanwhile, the majority of the transcripts were stimulated patched binding (GO:0005113) in the GO molecular function analysis and related to the condensed nuclear chromosome kinetochore (GO:0000778) in the GO cellular component analysis. In the KEGG pathway analysis, there were 125 pathways for which the DEGs were enriched (Supplementary Table S4), the most predominant pathways being the cell cycle, one carbon pool by folate, progesterone-mediated oocyte maturation, vitamin B6 metabolism, homologous recombination, oocyte meiosis, p53 signaling pathway, Fanconi anemia pathway, cellular senescence, and notch signaling pathway (Figure 2D). The co-expression network was constructed to investigate the potential interaction among DEGs in obese BC compared with the non-obese BC patients. The DEGs interacted with 46 genes by 2,863 total links (Figure 2E). Therefore, each DEG correlates with a large number of mRNA targets, suggesting that the interconnection between DEGs and mRNAs may related to obesity.

### Validation of the Transcriptomic Data via Investigated OLFM4 Expression Level in Large BC Cohort

**Figure 3:**
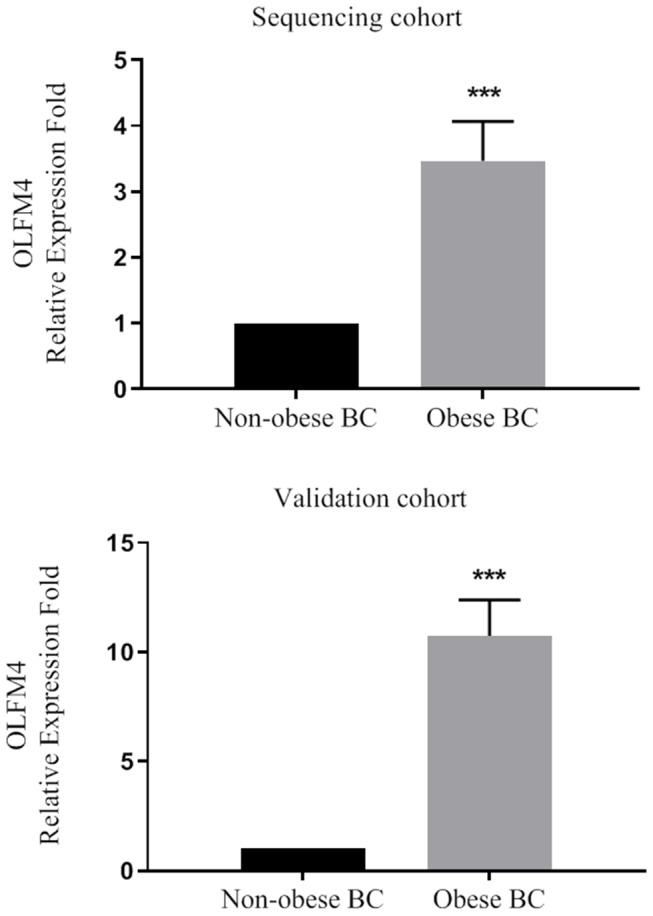
The OLFM4 expression level in the blood of obese BC compared with non-obese BC patients in the sequencing and validation cohort. The gene expression was detected by qRT-PCR and normalized by RPL11 expression; ***p < 0.0001.

Based on our RNA-seq data, the olfactomedin-4 (OLFM4) was chosen as it was among the most highly significant DEGs that altered. In addition, previous studies suggested a role of OLFM4 in immune cells and associate with increased risk of human cancers (39, 40). The expression level of OLFM4 was measured in a large validation cohort of BC patients (36 obese BC vs. 33 non-obese BC). From our RNA-seq data, the OLFM4 had an increased expression level in obese BC as compared with non-obese BC patients (3.47-folds; *p* < 0.0001). Moreover, the OLFM4 was still highly significantly upregulated (10.74-folds; *p* < 0.0001) in obese BC compared with non-obese BC patients in a large validation cohort (Figure 3). Therefore, the validation results showed concordance with the RNA-seq trend.

### Untargeted Metabolomics of Circulating Metabolites of Obese BC and Non-Obese BC Patients

**Figure 4:**
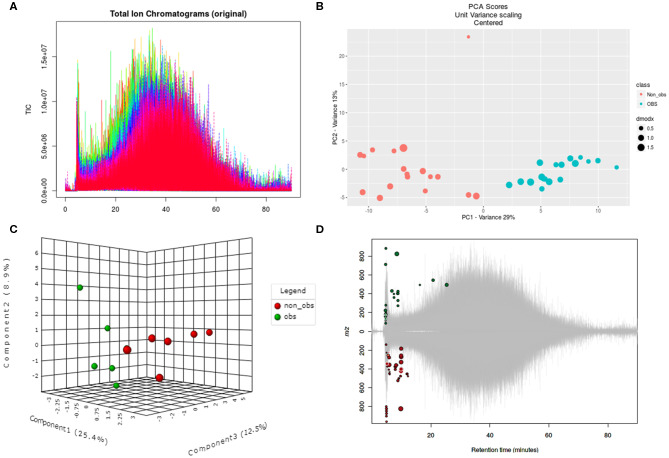
Untargeted metabolomics of obese and non-obese BC patients. **(A)** Total ion chromatogram of two groups in triplicates. **(B)** Two dimensional PCA score plot with experimental triplicate between samples. **(C)** Three-dimensional PLS-DA score plot between individual samples. **(D)** Significant metabolic features are marked in respective retention time and spot size indicates its abundance. PCA, principal component analysis; PLS-DA, partial least squares–discriminant analysis; OBS, obese BC.

**Figure 5:**
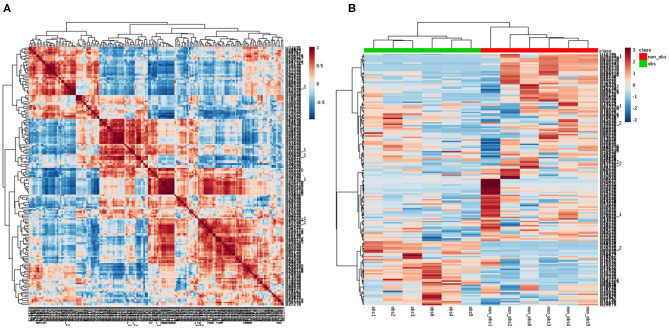
Correlation of the metabolomics data. **(A)** Each metabolite in the square represent the Spearman's correlation coefficient between (*r*^2^) are calculated between each metabolite across all metabolites. Metabolite order is determined as in hierarchical clustering. Self-correlations are identified in red. **(B)** HCA-heatmap analysis showing positive (red) and negative (blue) comparison metabolites of obese BC (green) and non-obese BC (red) patients. *P* ≤ 0.05 for all the metabolites OBS, obese BC.

We performed the metabolic study in obese BC patients and compared it with the non-obese BC, selected among the same RNA-seq patients using untargeted LC-MS/MS-based metabolomics. After analyzing the feature peaks, 173 features were detected in ESI+ mode. Two-dimensional principal component analysis (PCA) and three dimensional partial least squares-discriminant analysis (PLS-DA) models score plots of all samples showed no outliers in our study and revealed a significant difference in metabolomics between obese BC and non-obese BC samples (Figures 4A–D). We identified 173 metabolites, of which, 100 were downregulated while 73 metabolites were upregulated in obese BC compared with non-obese BC patients. Among these, 96 metabolites were significantly different (Supplementary Table S5), with 36 upregulated and 60 downregulated metabolites. The Spearman's correlation coefficient of the metabolomics data was evidenced by metabolites self-correlations (Figure 5A). However, in the hierarchical cluster analysis (HCA)-heatmap for the differential metabolites, the obese BC samples clustered and separated from non-obese BC (Figure 5B).

### Pathway Enrichment Analysis of Deregulated Circulating Metabolites

**Figure 6:**
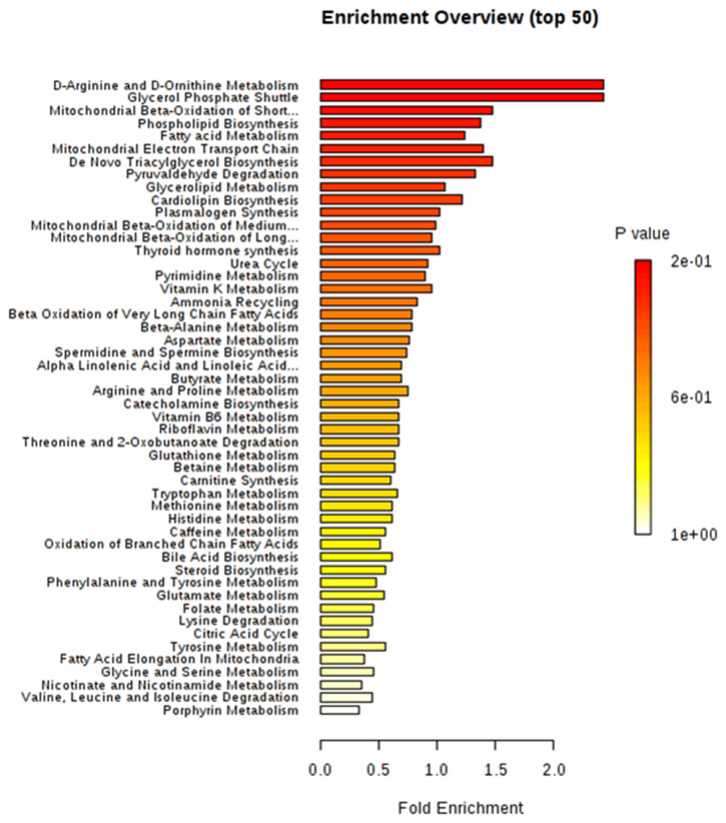
The top 50 enriched pathway analysis of significant differential accumulated metabolites between obese and non-obese BC patients.

The differential metabolites between obese BC and non-obese BC samples were used for pathway enrichment analysis. A total of 56 metabolic pathways were shown to be enriched in obese BC compared with non-obese BC patients (Supplementary Figure S1 and Supplementary Table S6), mainly involved in lipid, carbohydrate, and amino acid metabolism. As well as oxidative phosphorylation, and some other metabolic pathways, such as urea cycle, ammonia recycling, vitamins metabolism, etc. (Figure 6), which play important roles in ATP generation and cancer cell proliferation and metastasis, therefore, some can be utilized as novel therapeutic targets for cancer therapy (41).

The most important upregulated functionally metabolites were related to epigenetic as well as metabolic pathways that are involved in energy metabolism and cell proliferation such as amino acid and citric acid cycle. Furthermore, the upregulated neurotransmitters metabolites as serotonin, histamine, and acetylcholine may play a different role in the immune system (Table 4).

**Table 4:** Metabolites identified in obese BC compared with non-obese BC patients.

**Category**	**Compound**	**Log2FC**	***p*-value**
Metabolite involved in epigenetic	Ornithine	2.42	<0.0001
	7-Methyladenine	2.40	<0.0001
	2-Oxoarginine	1.74	<0.0001
Metabolite involved in citric acid cycle	L-Carnitine	1.83	<0.0001
	Glycerol 3-phosphate	2.38	<0.0001
	L-2-Hydroxyglutaric acid	0.71	0.0004
	Pentanoyl-CoA	−1.95	0.0001
Amino acid metabolism	Ornithine	2.42	<0.0001
	2-Oxoarginine	1.74	<0.0001
	Serotonin	0.73	0.0003
	Histamine	0.76	0.003
	Tryptophan	0.42	0.024
	L-Homoserine	0.44	0.038
	D-Leucine	2.34	<0.0001
	3-Hydroxyphenylacetic acid	−4.97	<0.0001
	Carbamoyl phosphate	−1.36	0.0001
	FAD	−2.28	0.0002
	Epinephrine	−0.33	0.0003
	Creatinine	−0.40	0.01
Cholesterol and fatty acid metabolites	25-Hydroxycholesterol	2.31	<0.0001
	Cholestenone	−7.85	<0.0001
	TG[16:0/14:1(9Z)/18:4(6Z,9Z,12Z,15Z)]	−1.52	0.0006
	Alpha-Linolenic acid	−0.77	0.002
	Hexanoyl-CoA	−1.71	0.0004
Neurotransmitters	Epinephrine	−0.33	0.0003
	Serotonin	0.73	0.0003
	SM[d19:1/24:1(15Z)]	6.55	0.0008
	Histamine	0.76	0.003
	Acetylcholine	0.55	0.007

*FC, fold change*.

### Integration Analysis of Transcriptomic and Metabolomic Data

**Figure 7:**
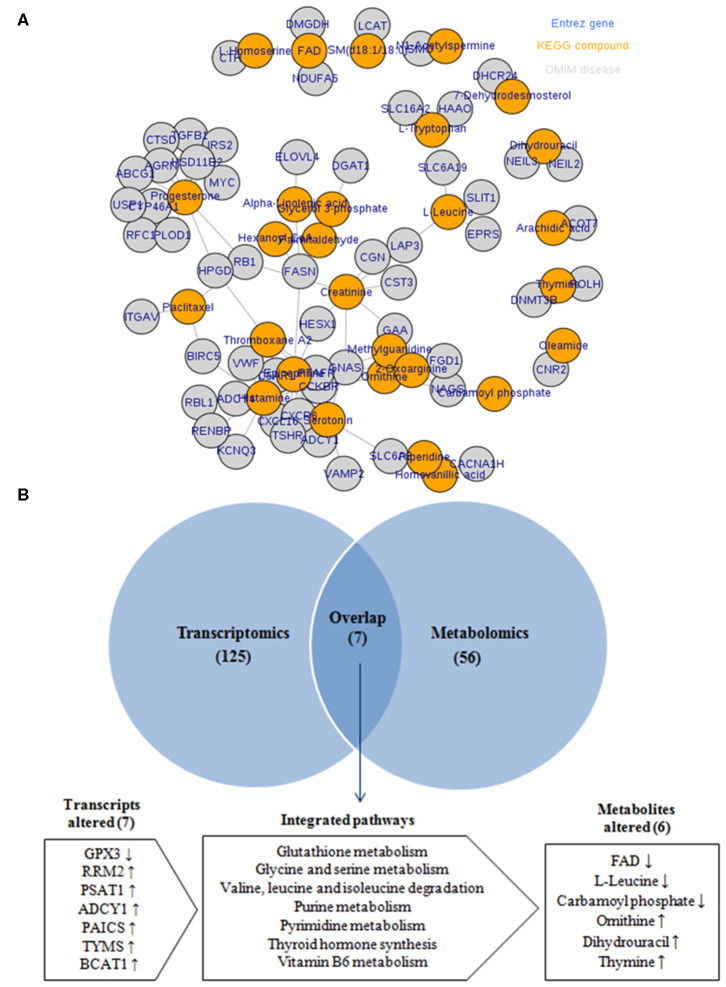
Integration of transcriptomic and metabolomic data. **(A)** The transcript–metabolite interaction network of the integration network consists of 65 nodes connected through 91 edges. Nodes in orange indicated differential metabolites and nodes in gray indicated DEGs related to each metabolite. **(B)** Venn diagram of the transcriptomic and metabolomic enriched pathway.

To provide more comprehensive understanding for the association between obesity and BC, the transcript–metabolite interaction network was generated for DEGs and the deregulated metabolites, in obese vs. non-obese BC samples. This provides a visualization of the interactions between functionally related metabolites and genes identified from transcriptomics and metabolomics. The gene–metabolite interaction consists of 65 nodes connected via 91 edges (Figure 7A). Furthermore, integration analysis at the pathway level was undertaken. Seven pathways were enriched during the integration of both transcriptomics and metabolomics data, that includes glutathione metabolism, glycine and serine metabolism, valine, leucine, and isoleucine degradation, purine metabolism, pyrimidine metabolism, thyroid hormone synthesis, and vitamin B6 metabolism (Figure 7B). The DEGs that relate to the integration pathways including RRM2, PSAT1, ADCY1, PAICS, TYMS, and BCAT1 were significantly upregulated, whereas the GPX3 gene was significantly downregulated. While, the differentially accumulated metabolites FAD, L-leucine, and carbamoyl phosphate, were significantly downregulated, whereas ornithine, dihydrouracil, and thymine metabolites, were significantly upregulated in obese compared to non-obese BC samples (Supplementary Table S7). Overall, the integration analysis successfully identified pathways and its related metabolites that can widely affect the functions of immune cells in obese BC patients.

## Discussion

Transcriptomics and metabolomics reflect changes in genotype and phenotype, respectively and provide complementary information about genetic alteration, protein synthesis, metabolisms and cellular function (42, 43). Many studies focused on the differential transcripts and metabolites and their functional attributes to understanding the disease's biological interaction. It has been revealed that differentially expressed genes that lead to bio-fluid metabolome change may significantly contribute to the initiation and progression of many types of diseases including obesity and BC (44–47). Currently, the most widely techniques that are used to differentiation in the transcriptomic and metabolomic profiles are RNA-seq, LC-MS/MS, respectively (48, 49). Over the last few years, several transcriptomic and metabolomic studies identified the variation in transcripts and metabolites profiles related with diseases such as obesity and BC compared with non-obese and healthy control cases, respectively, to understand the mechanisms of disease initiation and progression as well as to biomarker identification to deduce the onset and progression of cancer (16, 20, 21, 50–55). Furthermore, other studies have performed the integration between transcriptomic and metabolomic data (14, 56–58). Interestingly, the molecular mechanisms underlying the association between obesity and BC risk are not well-understood and still unclear (18). In an effort to reduce the knowledge gap, we performed these experiments to clarify the potential relationship between obesity and BC by the integration of the peripheral blood differential transcriptomic and metabolomic profiles at the pathway level. Noteworthy, to the best of our knowledge, the approach to our study between obese and non-obese BC patients has not been previously applied. Furthermore, the outcomes of the above-referred transcriptomics and metabolomics studies deals with healthy non-obese cases vs. obese patients, as well as with healthy control cases vs. BC patients were different from our findings of transcriptomics and metabolomics investigation as well as their integration, in obese compared with non-obese BC patients.

In this study, during the comparison of gene expression levels among obese and non-obese BC patients, we identified 2,272 significant DEGs, of which 1,737 transcripts were upregulated and 635 transcripts were downregulated in obese BC. GO analysis and co-expression networks of DEGs were performed to delineates the molecular mechanism and identify interactions among the discovered genes. Unique deregulated transcripts were enriched in different cellular pathways such as: cell cycle, one carbon pathway, homologous recombination cellular senescence, P53, and notch signaling pathway, in obese BC patients when compared with non-obese BC patients. Therefore, DEGs might essentially contribute to the initiation and/or development of obesity that may lead to BC initiation and/or progresses. These findings were different from previously reported studies; such as the Merdad et al., transcriptomics study in the tissue of BC patients compared with normal controls that observed downregulated genes associated with lipid metabolism pathway (20). Additional to Sun et al., transcriptomics study that reveled three deregulated long non-coding RNA (lncRNA) (lncRNA-p5549, lncRNA-p21015, and lncRNA-p19461) in the circulation of obese vs. non-obese individuals (51).

The expression pattern of OLFM4 has been differentially reported among tissues type (59). According to the human protein atlas, the OLFM4 was mainly expressed in the gastrointestinal tract, bone marrow, and immune system. Albuquerque et al. (60) demonstrated that OLFM4 was upregulated in obese children. Moreover, OLFM4 was also highly expressed in colon, breast, and lung cancerous tissues (61), where it inhibits apoptosis and promotes cancer cell proliferation, suggesting it may serve as a diagnostic marker or a therapeutic target for human cancers (39). Our findings revealed that OLFM4 was upregulated in the blood of obese BC as compared with non-obese BC patients. Therefore, the OLFM4 was upregulated in blood immune cells in obesity with BC, suggesting that it may play a unique role in immune cells and associated with increased risk of BC (39, 40).

On the other hand, untargeted metabolome analysis was performed in serum samples selected among the same RNA-seq patient populations. We detected a total of 96 circulating metabolites deregulated in obese BC patients when compared to non-obese BC patients, enriched in 56 metabolic pathways, mainly involved in cellular functions regulation and playing an important roles in ATP generation, cancer cell proliferation, and metastasis, therefore, it can be used as novel therapeutic agents for cancer therapy (41). Moreover, the most important upregulated metabolites that act as epigenetic, play an immunoregulatory role and involved in energy metabolism and cell proliferation.

Finally, the integration analysis between transcriptomic and metabolomic data at the pathway level provided a visualization of the interactions between DEGs and differentially metabolites. Collectively, deregulated obesity-associated genes and metabolites involved in changes of pathways, effective cancer cells metabolic programs, and increase BC risk (62). Data integration revealed novel seven uniquely enriched pathways in obese BC patients when compared with non-obese BC patients. The glutathione metabolism as one of the integrated metabolism, utilized FAD in the glutathione biosynthesis, which promotes tumor progression, metastasis, and protects cancer cells (63). Interestingly, downregulation of FAD metabolite and glutathione peroxidase 3 (GPX3) was detected in obese BC patients. GPX3 protein protects the cells against oxidative damage, thereby the low expression of GPX3 was associated with breast carcinogenesis (64). Free amino acids as glycine, serine, and branched-chain amino acid (BCAA) were associated with obesity and various types of cancer including BC. Glycine and serine metabolism provides the essential precursors for proteins and nucleic acid biosynthesis (65–67). The phosphoserine aminotransferase 1 (PSAT1) and branched-chain amino acid transaminase 1 (BCAT1) were upregulated in many carcinoma tissues and associated with cell proliferation (67, 68). PSAT1 and BCAT1, which played an important enzymatic role in serine metabolism and BCAA degradation, respectively, were found to be upregulated in obese BC patients. Ribonucleotide reductase regulatory subunit M2 (RRM2), an enzyme involved in dNTP production, increased dNTP pools and related with purine and pyrimidine metabolism (69) is tumorigenic and upregulated in cancer cells (70), was also found to be upregulated. The dysregulation of the purine metabolism pathway was also demonstrated in the integration of metabolomic and transcriptomic data of BC patients compared with healthy subjects, which might affect the BC progression (57). All these findings, could enhance the integration between transcripts and metabolomics and provided resistance for BC cell to dodge the circulating immune cells of whole blood.

In conclusion, our results demonstrate alteration in pathways at transcriptome and metabolome level in obese BC patients. This may suggest that obesity-associated transcripts and metabolites reveals alteration in metabolic pathway networks and rewire metabolic programs in cancer cells. This information could provide an important tool in research and may add to the knowledge on the molecular interaction between obesity and BC.

There are some limitations in this work, including a small sample size in metabolomic analysis. In addition, the quantity of blood RNA in samples depended mainly on the content of white blood cells. Therefore, the volume of our blood samples may contain limited amount of circulating RNAs. Further control cross-sectional studies using healthy obese and non-obese patients as well as increase in metabolomics sample size, will be conducted in the near future. Finally, the identification of OLFM4 expressing immune cells and the functional role of OLFM4 in immune cells need further investigation.

## Data Availability Statement

The original contributions presented in the study are publicly available. This data can be found here: the NCBI Gene Expression Omnibus (GSE148892).

## Ethics Statement

The studies involving human participants were reviewed and approved by the Unit of Biomedical Ethics, Research Committee (Document number: HA-02-J-008), King Abdulaziz University Hospital, King Abdulaziz University, Jeddah, Saudi Arabia. The patients/participants provided their written informed consent to participate in this study.

## Author Contributions

MH, HC, KA-S, AA-M, MM, and MK designed and coordinated the experiments. SA diagnosed and recruit the patients. KA-S, JA-M, and SA obtained ethical approval, patient consent, and provided the samples. MH, MS, and AD performed the experiments. JA-M performed the pathological screening. KA-S, HC, and AA contributed in laboratory facilitates. MH and MS analyzed metabolomics data. JR and WW analyzed transcriptomics data. MH wrote the original manuscript draft. HC, AA, MK, and MM edited the manuscript. KA-S, AA, SA, HC, and JA-M provided project funding. All authors read and approved the final manuscript.

## Conflict of Interest

The authors declare that the research was conducted in the absence of any commercial or financial relationships that could be construed as a potential conflict of interest.

## References

[1] DeSantis CE Bray F Ferlay J Lortet-Tieulent J Anderson BO Jemal A. International variation in female breast cancer incidence and mortality rates. Cancer Epidemiol Biomark Prev. (2015) 24:1495–506. 10.1158/1055-9965.EPI-15-0535 26359465

[2] Bazarbashi S Al Eid H Minguet J. Cancer incidence in Saudi Arabia: 2012 data from the Saudi Cancer registry. Asian Pac J Cancer Prev. (2017) 18:2437–44. 10.22034/APJCP.2017.18.9.2437 28952273 PMC5720648

[3] Bray F Ferlay J Soerjomataram I Siegel RL Torre LA Jemal A. Global cancer statistics 2018: GLOBOCAN estimates of incidence and mortality worldwide for 36 cancers in 185 countries. CA Cancer J Clin. (2018) 68:394–424. 10.3322/caac.21492 30207593

[4] Eroles P Bosch A Perez-Fidalgo JA Lluch A. Molecular biology in breast cancer: intrinsic subtypes and signaling pathways. Cancer Treat Rev. (2012) 38:698–707. 10.1016/j.ctrv.2011.11.005 22178455

[5] Rudolph A Chang-Claude J Schmidt MK. Gene-environment interaction and risk of breast cancer. Br J Cancer. (2016) 114:125–33. 10.1038/bjc.2015.439 26757262 PMC4815812

[6] Smith KB Smith MS. Obesity statistics. Primary Care. (2016) 43:121–35. 10.1016/j.pop.2015.10.001 26896205

[7] Memish ZA El Bcheraoui C Tuffaha M Robinson M Daoud F Jaber S. Obesity and associated factors–Kingdom of Saudi Arabia, 2013. Prev Chronic Dis. (2014) 11:E174. 10.5888/pcd11.140236 25299980 PMC4193060

[8] Lorincz AM Sukumar S. Molecular links between obesity and breast cancer. Endocr Relat Cancer. (2006) 13:279–92. 10.1677/erc.1.00729 16728564

[9] Abulkhair O Dietary Fat, Obesity, Estrogen level and breast cancer risk in Saudi female: a case-control study. Cancer Res. (2009) 69(24 Suppl):6070 10.1158/0008-5472.SABCS-09-6070

[10] Elkum N Al-Tweigeri T Ajarim D Al-Zahrani A Amer SMB Aboussekhra A. Obesity is a significant risk factor for breast cancer in Arab women. BMC Cancer. (2014) 14:788. 10.1186/1471-2407-14-788 25351244 PMC4532295

[11] McPherson K Steel CM Dixon JM. ABC of breast diseases. Breast cancer-epidemiology, risk factors, and genetics. BMJ. (2000) 321:624–8. 10.1136/bmj.321.7261.624 10977847 PMC1118507

[12] Amadou A Ferrari P Muwonge R Moskal A Biessy C Romieu I. Overweight, obesity and risk of premenopausal breast cancer according to ethnicity: a systematic review and dose-response meta-analysis. Obesity Rev. (2013) 14:665–78. 10.1111/obr.12028 23615120

[13] Calle EE Rodriguez C Walker-Thurmond K Thun MJ. Overweight, obesity, and mortality from cancer in a prospectively studied cohort of U.S. adults. N Engl J Med. (2003) 348:1625–38. 10.1056/NEJMoa021423 12711737

[14] Cavill R Jennen D Kleinjans J Briede JJ. Transcriptomic and metabolomic data integration. Brief Bioinform. (2016) 17:891–901. 10.1093/bib/bbv090 26467821

[15] Casamassimi A Federico A Rienzo M Esposito S Ciccodicola A. Transcriptome profiling in human diseases: new advances and perspectives. Int J Mol Sci. (2017) 18:E1652. 10.3390/ijms18081652 28758927 PMC5578042

[16] Weisman PS Ng CK Brogi E Eisenberg RE Won HH Piscuoglio S. Genetic alterations of triple negative breast cancer by targeted next-generation sequencing and correlation with tumor morphology. Mod Pathol. (2016) 29:476–88. 10.1038/modpathol.2016.39 26939876 PMC4848211

[17] Xu J Zhao R Xue Y Xiao H Sheng Y Zhao D. RNA-seq profiling reveals differentially expressed genes as potential markers for vital reaction in skin contusion: a pilot study. Forensic Sci Res. (2018) 3:153–60. 10.1080/20961790.2017.1349639 30483664 PMC6197083

[18] Mamidi TKK Wu J Tchounwou PB Miele L Hicks C. Whole genome transcriptome analysis of the association between obesity and triple-negative breast cancer in caucasian women. Int J Environ Res Public Health. (2018) 15:E2338. 10.3390/ijerph15112338 30360534 PMC6265882

[19] Wu W Choudhry H (editors). Next Generation Sequencing in Cancer Research. Vol. 2, From Basepairs to Bedsides. Cham: Springer (2015).

[20] Merdad A Karim S Schulten HJ Jayapal M Dallol A Buhmeida A. Transcriptomics profiling study of breast cancer from Kingdom of Saudi Arabia revealed altered expression of Adiponectin and Fatty Acid Binding Protein4: Is lipid metabolism associated with breast cancer? BMC Genomics. (2015) 16(Suppl 1):S11. 10.1186/1471-2164-16-S1-S11 25923423 PMC4315151

[21] Yin P Xu G. Metabolomics for tumor marker discovery and identification based on chromatography-mass spectrometry. Expert Rev Mol Diagn. (2013) 13:339–48. 10.1586/erm.13.23 23638817

[22] Ament Z Masoodi M Griffin JL. Applications of metabolomics for understanding the action of peroxisome proliferator-activated receptors (PPARs) in diabetes, obesity and cancer. Genome Med. (2012) 4:32. 10.1186/gm331 22546357 PMC3446260

[23] de Onis M Habicht JP. Anthropometric reference data for international use: recommendations from a World Health Organization Expert Committee. Am J Clin Nutr. (1996) 64:650–8. 10.1093/ajcn/64.4.650 8839517

[24] Razvi SS Choudhry H Hasan MN Hassan MA Moselhy SS Abualnaja KO. Identification of deregulated signaling pathways in jurkat cells in response to a novel acylspermidine analogue-N4-erucoyl spermidine. Epigenet Insights. (2018) 11:2516865718814543. 10.1177/2516865718814543 30515476 PMC6262497

[25] Gordon A Hannon G Fastx-toolkit. FASTQ/A Short-reads Pre-processing Tools. (2010). Available online at: http://hannonlab.cshl.edu/fastx_toolkit

[26] Kim D Pertea G Trapnell C Pimentel H Kelley R Salzberg SL. TopHat2: accurate alignment of transcriptomes in the presence of insertions, deletions and gene fusions. Genome Biol. (2013) 14:R36. 10.1186/gb-2013-14-4-r36 23618408 PMC4053844

[27] Liao Y Smyth GK Shi W. featureCounts: an efficient general purpose program for assigning sequence reads to genomic features. Bioinformatics. (2013) 30:923–30. 10.1093/bioinformatics/btt656 24227677

[28] Robinson MD McCarthy DJ Smyth GK. edgeR: a Bioconductor package for differential expression analysis of digital gene expression data. Bioinformatics. (2010) 26:139–40. 10.1093/bioinformatics/btp616 19910308 PMC2796818

[29] Ashburner M Ball CA Blake JA Botstein D Butler H Cherry JM. Gene ontology: tool for the unification of biology. Nat Genet. (2000) 25:25–9. 10.1038/75556 10802651 PMC3037419

[30] Kuleshov MV Jones MR Rouillard AD Fernandez NF Duan Q Wang Z. Enrichr: a comprehensive gene set enrichment analysis web server 2016 update. Nucleic Acids Res. (2016) 44:W90–W7. 10.1093/nar/gkw377 27141961 PMC4987924

[31] Kanehisa M Goto S. KEGG: kyoto encyclopedia of genes and genomes. Nucleic Acids Res. (2000) 28:27–30. 10.1093/nar/28.1.27 10592173 PMC102409

[32] Warde-Farley D Donaldson SL Comes O Zuberi K Badrawi R Chao P. The GeneMANIA prediction server: biological network integration for gene prioritization and predicting gene function. Nucleic Acids Res. (2010) 38:W214–W20. 10.1093/nar/gkq537 20576703 PMC2896186

[33] Livak KJ Schmittgen TD. Analysis of relative gene expression data using real-time quantitative PCR and the 2^−ΔΔCT^ method. Methods. (2001) 25:402–8. 10.1006/meth.2001.1262 11846609

[34] Nadeem MS Razeeth M Choudhry HMZ Anwar F Zamzami MA Murtaza BN. LC-MS/MS-based metabolic profiling of *Escherichia coli* under heterologous gene expression stress. J Cell Biochem. (2019) 121:125–34. 10.1002/jcb.28962 31232490

[35] Smith CA Want EJ O'Maille G Abagyan R Siuzdak G. XCMS: processing mass spectrometry data for metabolite profiling using nonlinear peak alignment, matching, and identification. Anal Chem. (2006) 78:779–87. 10.1021/ac051437y 16448051

[36] Kuhl C Tautenhahn R Bottcher C Larson TR Neumann S. CAMERA: an integrated strategy for compound spectra extraction and annotation of liquid chromatography/mass spectrometry data sets. Anal Chem. (2012) 84:283–9. 10.1021/ac202450g 22111785 PMC3658281

[37] Smith CA O'Maille G Want EJ Qin C Trauger SA Brandon TR. METLIN: a metabolite mass spectral database. Ther Drug Monit. (2005) 27:747–51. 10.1097/01.ftd.0000179845.53213.39 16404815

[38] Chong J Xia J. MetaboAnalystR: an R package for flexible and reproducible analysis of metabolomics data. Bioinformatics. (2018) 34:4313–4. 10.1093/bioinformatics/bty528 29955821 PMC6289126

[39] Liu W Rodgers GP. Olfactomedin 4 expression and functions in innate immunity, inflammation, and cancer. Cancer Metastasis Rev. (2016) 35:201–12. 10.1007/s10555-016-9624-2 27178440

[40] Alder MN Mallela J Opoka AM Lahni P Hildeman DA Wong HR. Olfactomedin 4 marks a subset of neutrophils in mice. Innate Immun. (2019) 25:22–33. 10.1177/1753425918817611 30537894 PMC6661892

[41] Lukey MJ Katt WP Cerione RA. Targeting amino acid metabolism for cancer therapy. Drug DisToday. (2017) 22:796–804. 10.1016/j.drudis.2016.12.003 27988359 PMC5429979

[42] Liu JL Zhang WQ Zhao M Huang MY. Integration of transcriptomic and metabolomic data reveals enhanced steroid hormone biosynthesis in mouse uterus during decidualization. Proteomics. (2017) 17:1700059. 10.1002/pmic.201700059 28857456

[43] Zhang G He P Tan H Budhu A Gaedcke J Ghadimi BM. Integration of metabolomics and transcriptomics revealed a fatty acid network exerting growth inhibitory effects in human pancreatic cancer. Clin Cancer Res. (2013) 19:4983–93. 10.1158/1078-0432.CCR-13-0209 23918603 PMC3778077

[44] Rodriguez-Esteban R Jiang X. Differential gene expression in disease: a comparison between high-throughput studies and the literature. BMC Med Genomics. (2017) 10:59. 10.1186/s12920-017-0293-y 29020950 PMC5637346

[45] Dermitzakis ET. From gene expression to disease risk. Nat Genet. (2008) 40:492–3. 10.1038/ng0508-492 18443581

[46] Güler EN. Gene expression profiling in breast cancer and its effect on therapy selection in early-stage breast cancer. Eur J Breast Health. (2017) 13:168–74. 10.5152/ejbh.2017.3636 29082373 PMC5648272

[47] Fan Y Zhou X Xia TS Chen Z Li J Liu Q. Human plasma metabolomics for identifying differential metabolites and predicting molecular subtypes of breast cancer. Oncotarget. (2016) 7:9925–38. 10.18632/oncotarget.7155 26848530 PMC4891093

[48] Ritchie ME Phipson B Wu D Hu Y Law CW Shi W. limma powers differential expression analyses for RNA-sequencing and microarray studies. Nucleic Acids Res. (2015) 43:e47. 10.1093/nar/gkv007 25605792 PMC4402510

[49] Chaleckis R Meister I Zhang P Wheelock CE. Challenges, progress and promises of metabolite annotation for LC-MS-based metabolomics. Curr Opin Biotechnol. (2019) 55:44–50. 10.1016/j.copbio.2018.07.010 30138778

[50] Cirulli ET Guo L Leon Swisher C Shah N Huang L Napier LA. Profound perturbation of the metabolome in obesity is associated with health risk. Cell Metab. (2019) 29:488–500.e2. 10.1016/j.cmet.2018.09.022 30318341 PMC6370944

[51] Sun J Ruan Y Wang M Chen R Yu N Sun L. Differentially expressed circulating LncRNAs and mRNA identified by microarray analysis in obese patients. Sci Rep. (2016) 6:35421. 10.1038/srep35421 27767123 PMC5073332

[52] Long T Hicks M Yu HC Biggs WH Kirkness EF Menni C. Whole-genome sequencing identifies common-to-rare variants associated with human blood metabolites. Nat Genet. (2017) 49:568–78. 10.1038/ng.3809 28263315

[53] Reinehr T Wolters B Knop C Lass N Hellmuth C Harder U. Changes in the serum metabolite profile in obese children with weight loss. Eur J Nutr. (2015) 54:173–81. 10.1007/s00394-014-0698-8 24740590

[54] Chen Z Li Z Li H Jiang Y. Metabolomics: a promising diagnostic and therapeutic implement for breast cancer. OncoTargets Ther. (2019) 12:6797–811. 10.2147/OTT.S215628 31686838 PMC6709037

[55] Shen J Yan L Liu S Ambrosone CB Zhao H. Plasma metabolomic profiles in breast cancer patients and healthy controls: by race and tumor receptor subtypes. Transl Oncol. (2013) 6:757–65. 10.1593/tlo.13619 24466379 PMC3890711

[56] Yang K Xia B Wang W Cheng J Yin M Xie H. A comprehensive analysis of metabolomics and transcriptomics in cervical cancer. Sci Rep. (2017) 7:43353. 10.1038/srep43353 28225065 PMC5320559

[57] Luo X Yu H Song Y Sun T. Integration of metabolomic and transcriptomic data reveals metabolic pathway alteration in breast cancer and impact of related signature on survival. J Cell Physiol. (2018) 234:13021–31. 10.1002/jcp.27973 30556899 PMC13483261

[58] Ren S Shao Y Zhao X Hong CS Wang F Lu X. Integration of metabolomics and transcriptomics reveals major metabolic pathways and potential biomarker involved in prostate cancer. Mol Cell Proteomics. (2016) 15:154–63. 10.1074/mcp.M115.052381 26545398 PMC4762514

[59] Wang XY Chen SH Zhang YN Xu CF. Olfactomedin-4 in digestive diseases: a mini-review. World J Gastroenterol. (2018) 24:1881–7. 10.3748/wjg.v24.i17.1881 29740203 PMC5937205

[60] Albuquerque D Nobrega C Rodriguez-Lopez R Manco L. Association study of common polymorphisms in MSRA, TFAP2B, MC4R, NRXN3, PPARGC1A, TMEM18, SEC16B, HOXB5 and OLFM4 genes with obesity-related traits among Portuguese children. J Hum Genet. (2014) 59:307–13. 10.1038/jhg.2014.23 24670271

[61] Koshida S Kobayashi D Moriai R Tsuji N Watanabe N. Specific overexpression of OLFM4GW112/hGC−1 mRNA in colon, breast and lung cancer tissues detected using quantitative analysis. Cancer Sci. (2007) 98:315–20. 10.1111/j.1349-7006.2006.00383.x 17270020 PMC11159027

[62] Madak-Erdogan Z Band S Zhao YC Smith BP Kulkoyluoglu-Cotul E Zuo Q. Free fatty acids rewire cancer metabolism in obesity-associated breast cancer via estrogen receptor and mTOR signaling. Cancer Res. (2019) 79:2494–510. 10.1158/0008-5472.CAN-18-2849 30862719

[63] Bansal A Simon MC. Glutathione metabolism in cancer progression and treatment resistance. J Cell Biol. (2018) 217:2291–8. 10.1083/jcb.201804161 29915025 PMC6028537

[64] Mohamed MM Sabet S Peng D-F Nouh MA El-Shinawi M El-Rifai W. Promoter hypermethylation and suppression of glutathione peroxidase 3 are associated with inflammatory breast carcinogenesis. Oxid Med Cell Longev. (2014) 2014:787195. 10.1155/2014/787195 24790704 PMC3980917

[65] Alves A Bassot A Bulteau A-L Pirola L Morio B. Glycine metabolism and its alterations in obesity and metabolic diseases. Nutrients. (2019) 11:1356. 10.3390/nu11061356 31208147 PMC6627940

[66] Amelio I Cutruzzolá F Antonov A Agostini M Melino G. Serine and glycine metabolism in cancer. Trends Biochem Sci. (2014) 39:191–8. 10.1016/j.tibs.2014.02.004 24657017 PMC3989988

[67] Siddik MAB Shin AC. Recent progress on branched-chain amino acids in obesity, diabetes, and beyond. Endocrinol Metab. (2019) 34:234–46. 10.3803/EnM.2019.34.3.234 31565875 PMC6769348

[68] Liu B Jia Y Cao Y Wu S Jiang H Sun X. Overexpression of phosphoserine aminotransferase 1 (PSAT1) predicts poor prognosis and associates with tumor progression in human esophageal squamous cell carcinoma. Cell Physiol Biochem. (2016) 39:395–406. 10.1159/000445633 27372650

[69] D'Angiolella V Donato V Forrester FM Jeong Y-T Pellacani C Kudo Y. Cyclin F-mediated degradation of ribonucleotide reductase M2 controls genome integrity and DNA repair. Cell. (2012) 149:1023–34. 10.1016/j.cell.2012.03.043 22632967 PMC3616325

[70] Buj R Aird KM. Deoxyribonucleotide triphosphate metabolism in cancer and metabolic disease. Front Endocrinol. (2018) 9:177. 10.3389/fendo.2018.00177 29720963 PMC5915462

