# Supplementary material for: Integration of Transcriptome and Metabolome Provides Unique Insights to Pathways Associated With Obese Breast Cancer Patients
Source: Front Oncol. 2020 May 19;10:804. doi: 10.3389/fonc.2020.00804 (PMC7248369; doi:10.3389/fonc.2020.00804)
Supplement: Supplementary file 1 [file Table_1.DOCX]

**Supplementary Table S1.** Distribution of general information characteristics of the study BC patients. Data were presented as total number N (%).

|  | **Parameters** | **Total**  **N (%)** | **Non-obese BC**  **N (%)** | **Obese BC**  **N (%)** | ***p*-value** |
| --- | --- | --- | --- | --- | --- |
|  |  |  |  |  |  |
| No. of patients |  | 21 (100) | 10 (47.6) | 11 (52.4) |  |
| Age of patients (years) | ≤40 | 4 (19.0) | 1 (25.0) | 3.0 (75.0) | 0.33 |
|  | 41-60 | 16 (76.2) | 9 (56.3) | 7.0 (43.8) |  |
|  | ˃60 | 1 (4.8) | 0.0 (0.0) | 1 (100) |  |
| Marital status | Single | 3 (14.3) | 1.0 (33.3) | 2.0 (66.7) | 0.51 |
|  | Married | 17 (81.0) | 8.0 (47.1) | 9.0 (52.9) |  |
|  | Divorce | 1 (4.8) | 1.0 (100.0) | 0.0 (0.0) |  |
| Educational level | Illiterate | 3 (14.3) | 2.0 (66.7) | 1.0 (33.3) | 0.70 |
|  | School | 11 (52.4) | 4.0 (36.4) | 7.0 (63.6) |  |
|  | First degree | 4 (19.0) | 2.0 (50.0) | 2.0 (50.0) |  |
|  | Higher degree | 3 (14.3) | 2.0 (66.7) | 1.0 (33.3) |  |
| Nationality | Saudi | 14 (66.7) | 6.0 (42.9) | 8.0 (57.1) | 0.54 |
|  | Non-Saudi | 7 (33.3) | 4.0 (57.1) | 3.0 (42.9) |  |
| Age of first menstruation (years) | ˂ 12 | 2 (9.5) | 2.0 (100.0) | 0.0 (0.0) | 0.20 |
|  | 12 - 15 | 18 (85.7) | 8.0 (44.4) | 10.0 (55.6) |  |
|  | > 15 | 1 (4.8) | 0.0 (0.0) | 1.0 (100.0) |  |
| Menopausal status | Postmenopausal | 8 (38.1) | 2.0 (25.0) | 6.0 (75.0) | 0.10 |
|  | Premenopausal | 13 (61.9) | 8.0 (61.5) | 5.0 (38.5) |  |
| Age of menopause (years) | ˂ 48 | 2 (25.0) | 1.0 (50.0) | 1.0 (50.0) | 0.35 |
|  | 48 - 55 | 6 (75.0) | 1.0 (16.7) | 5.0 (83.3) |  |
|  | > 55 | 0 (0.0) | 0.0 (0.0) | 0.0 (0.0) |  |
| Hormone replacement therapy (HRT) | Yes | 1 (4.8) | 0.0 (0.0) | 0.0 (0.0) | 0.33 |
|  | No | 20 (95.2) | 10.0 (50.0) | 10.0 (50.0) |  |
| Number of children | None | 2 (14.3) | 1.0 (50.0) | 1.0 (50.0) | 0.73 |
|  | 3 or less | 3 (21.4) | 1.0 (33.3) | 2.0 (66.7) |  |
|  | 4 to 6 | 7 (50.0) | 2.0 (28.6) | 5.0 (71.4) |  |
|  | more than 6 | 2 (14.3) | 0.0 (0.0) | 2.0 (100.0) |  |
| Number of miscarriages | None | 5 (35.7) | 3.0 (60.0) | 2.0 (40.0) | 0.63 |
|  | 1 & 2 | 6 (42.9) | 4.0 (66.7) | 2.0 (33.3) |  |
|  | 3 and more | 3 (21.4) | 1.0 (33.3) | 2.0 (66.7) |  |
| Age of pregnancy (years) | ≤ 20 | 3 (17.6) | 1.0 (33.3) | 3.0 (100.0) | 0.54 |
|  | 21 - 30 | 11 (64.7) | 5.0 (45.5) | 6.0 (54.5) |  |
|  | > 30 | 3 (17.6) | 2.0 (66.7) | 1.0 (33.3) |  |
| Breast feeding | Never | 4 (20.0) | 2 (50.0) | 2 (50.0) | 0.82 |
|  | Yes | 16 (80.0) | 7 (43.8) | 9 (56.3) |  |
| Family history of BC | Yes | 3 (15.8) | 2 (66.7) | 1 (33.3) | 0.35 |
|  | No | 16 (84.2) | 6 (37.5) | 10 (62.5) |  |
| Family history of other cancer | Yes | 3 (15.8) | 1 (33.3) | 2 (66.7) | 0.74 |
|  | No | 16 (84.2) | 7 (43.8) | 9 (56.3) |  |
| Polycystic fibrosis status | Yes | 4 (20.0) | 2 (50.0) | 2 (50.0) | 0.82 |
|  | No | 16 (80.0) | 7 (43.8) | 9 (56.3) |  |
| Diabetes mellitus status | Yes | 2 (10.0) | 1 (50.0) | 1 (50.0) | 0.88 |
|  | No | 18 (90.0) | 8 (44.4) | 10 (55.6) |  |
| Suffered from any other condition | Yes | 5 (26.3) | 1 (20.0) | 4 (80.0) | 0.24 |
|  | No | 14 (73.7) | 7 (50.0) | 7 (50.0) |  |
| Physical activities performance | Yes | 8 (38.1) | 4 (50.0) | 4 (50.0) | 0.86 |
|  | No | 13 (61.9) | 6 (46.2) | 7 (53.8) |  |
| Smoking | Yes | 2 (9.5) | 1 (50.0) | 1 (50.0) | 0.94 |
|  | No | 19 (90.5) | 9 (47.4) | 10 (52.6) |  |
| Omega 3 supplements | Yes | 5 (23.8) | 2 (40.0) | 3 (60.0) | 0.70 |
|  | No | 16 (76.2) | 8 (50.0) | 8 (50.0) |  |
| Fat rich diet | Yes | 7 (33.3) | 1 (14.3) | 6 (85.7) | 0.03 |
|  | No | 14 (66.7) | 9 (64.3) | 5 (35.7) |  |
