# Supplementary material for: Integration of Transcriptome and Metabolome Provides Unique Insights to Pathways Associated With Obese Breast Cancer Patients
Source: Front Oncol. 2020 May 19;10:804. doi: 10.3389/fonc.2020.00804 (PMC7248369; doi:10.3389/fonc.2020.00804)
Supplement: Supplementary file 2 [file Table_2.DOCX]

**Supplementary Table S2.** Distribution of clinicopathological characteristics of the study BC patients. Data were presented as total number N (%).

| **Parameters** | **Categories** | **Total**  **N (%)** | **Non-obese BC**  **N (%)** | **Obese BC**  **N (%)** | ***p*-value** |
| --- | --- | --- | --- | --- | --- |
| Number of patients |  | 21 (100) | 10 (47.6) | 11 (52.4) |  |
| Hormone receptor phenotype | Luminal | 15 (75.0) | 7 (46.7) | 8 (53.3) | 0.33 |
|  | HER2-enriched | 3 (15.0) | 2 (66.7) | 1 (33.3) |  |
|  | Triple negative/ basal like | 2 (10.0) | 0 (0.0) | 2 (100.0) |  |
|  | Unknown | 1 (4.8) | 1 (100) | 0 (0.0) |  |
| ER status | ER- | 5 (23.8) | 2 (40.0) | 3 (60.0) | 0.80 |
|  | ER+ | 15 (71.4) | 7 (46.7) | 8 (53.3) |  |
|  | Unknown | 1 (4.8) | 1 (100) | 0 (0.0) |  |
| PR status | PR- | 5 (23.8) | 2 (40.0) | 3 (60.0) | 0.80 |
|  | PR+ | 15 (71.4) | 7 (46.7) | 8 (53.3) |  |
|  | Unknown | 1 (4.8) | 1 (100) | 0 (0.0) |  |
| HER2 status | HER2- | 15 (71.4) | 6 (40.0) | 9 (60.0) | 0.44 |
|  | HER2+ | 5 (23.8) | 3 (60.0) | 2 (40.0) |  |
|  | Unknown | 1 (4.8) | 1 (100) | 0 (0.0) |  |
| Lymph node involvement | Negative | 11 (52.4) | 4 (36.4) | 7 (63.6) | 0.59 |
|  | Positive | 6 (28.6) | 3 (50.0) | 3 (50.0) |  |
|  | Unknown | 4 (19.0) | 3 (75.0) | 1 (25.0) |  |
| E-cadherin | Negative | 2 (9.5) | 1 (50.0) | 1 (50.0) | 0.25 |
|  | Positive | 2 (9.5) | 0 (0.0) | 2 (100.0) |  |
|  | Unknown | 17 (81.0) | 9 (52.9) | 8 (47.1) |  |
| Size of tumor (cm) | < 2 | 10 (47.6) | 6 (60.0) | 4 (40.0) | 0.23 |
|  | 2-5 | 10 (47.6) | 3 (30.0) | 7 (70.0) |  |
|  | > 5 | 1 (4.8) | 1 (100) | 0 (0.0) |  |
| Tumor grade | I | 4 (19.0) | 1 (25.0) | 3 (75.0) | 0.34 |
|  | II | 12 (57.1) | 5 (41.7) | 7 (58.3) |  |
|  | III | 4 (19.0) | 3 (75.0) | 1 (25.0) |  |
|  | Unknown | 1 (4.8) | 1 (100) | 0 (0.0) |  |
| Histotype | DCIS | 14 (66.7) | 5 (35.7) | 9 (64.3) | 0.12 |
|  | LCIS | 3 (14.3) | 1(33.3) | 2 (66.7) |  |
|  | Mixture of ductal and lobular | 3 (14.3) | 3 (100) | 0 (0.0) |  |
|  | Unknown | 1 (4.8) | 1 (100) | 0 (0.0) |  |
| Vascular invasion | Negative | 13 (61.9) | 7 (53.8) | 6 (46.2) | 0.13 |
|  | Positive | 6 (28.6) | 1 (16.7) | 5 (83.3) |  |
|  | Unknown | 2 (9.5) | 2 (100) | 0 (0.0) |  |
| Margin | Negative | 15 (71.4) | 6 (40.0) | 9 (60.0) | 0.42 |
|  | Positive | 1 (16.7) | 0 (0.0) | 1 (100.0) |  |
|  | Unknown | 5 (23.8) | 4 (80.0) | 1 (20.0) |  |
