# Supplementary material for: Integration of Transcriptome and Metabolome Provides Unique Insights to Pathways Associated With Obese Breast Cancer Patients
Source: Front Oncol. 2020 May 19;10:804. doi: 10.3389/fonc.2020.00804 (PMC7248369; doi:10.3389/fonc.2020.00804)
Supplement: Supplementary file 3 [file Table_3.DOCX]

**Supplementary Table S3.** The high significantly differentially expressed genes in obese compared with non-obese BC patients, ordered depending on logFC. LogFC rounded 2 numbers after the decimal point, FC: Fold change.

|  | **Gene symbol** | **logFC** | ***p*-value** |
| --- | --- | --- | --- |
| 1 | CHRNA3 | 6.98 | 0.0004 |
| 2 | SIX6 | 6.36 | 0.0009 |
| 3 | DPYSL5 | 6.24 | 0.0010 |
| 4 | ONECUT2 | 6.17 | 0.0007 |
| 5 | PEX5L | 5.65 | 0.0015 |
| 6 | FAT1 | 5.62 | 0.0011 |
| 7 | NDST3 | 5.61 | 0.0006 |
| 8 | ADCY1 | 5.52 | 0.0001 |
| 9 | PRAME | 5.52 | 0.0009 |
| 10 | PNMA2 | 5.44 | 0.0006 |
| 11 | DPF1 | 5.43 | 0.0014 |
| 12 | CRNDE | 5.28 | 0.0010 |
| 13 | HSPA4L | 4.84 | 0.0005 |
| 14 | THY1 | 4.75 | 0.0012 |
| 15 | SLC8A2 | 4.72 | 0.0008 |
| 16 | RADIL | 4.65 | 0.0007 |
| 17 | SH2D5 | 4.45 | 0.0004 |
| 18 | KIAA1549 | 4.41 | 0.0005 |
| 19 | ASPM | 4.33 | ˂ 0.0001 |
| 20 | COLEC11 | 4.31 | 0.0005 |
| 21 | E2F7 | 4.18 | 0.0001 |
| 22 | PXDN | 4.13 | 0.0004 |
| 23 | GALNT9 | 4.10 | 0.0001 |
| 24 | COL27A1 | 4.08 | 0.0002 |
| 25 | MYH10 | 4.03 | ˂ 0.0001 |
| 26 | 3-Sep | 3.90 | 0.0001 |
| 27 | CDK1 | 3.84 | 0.0001 |
| 28 | KIF14 | 3.81 | 0.0006 |
| 29 | STOX2 | 3.80 | 0.0008 |
| 30 | BEX1 | 3.74 | 0.0004 |
| 31 | AP001429.1 | 3.74 | ˂ 0.0001 |
| 32 | RASAL1 | 3.73 | 0.0006 |
| 33 | EPB41L1 | 3.72 | 0.0011 |
| 34 | IGFBP2 | 3.68 | 0.0001 |
| 35 | ILDR2 | 3.57 | 0.0010 |
| 36 | DEPDC1 | 3.56 | 0.0012 |
| 37 | ENAH | 3.52 | 0.0004 |
| 38 | TSPAN7 | 3.52 | 0.0013 |
| 39 | CPXM1 | 3.51 | 0.0002 |
| 40 | BUB1B | 3.50 | ˂ 0.0001 |
| 41 | HMMR | 3.49 | 0.0007 |
| 42 | CENPF | 3.49 | ˂ 0.0001 |
| 43 | OLFM4 | 3.47 | ˂ 0.0001 |
| 44 | TANC1 | 3.45 | 0.0011 |
| 45 | LRRC34 | 3.44 | 0.0002 |
| 46 | TOP2A | 3.44 | ˂ 0.0001 |
| 47 | TICRR | 3.42 | 0.0001 |
| 48 | ARMC9 | 3.36 | 0.0003 |
| 49 | MKRN3 | 3.35 | 0.0007 |
| 50 | TUB | 3.33 | 0.0011 |
| 51 | NUF2 | 3.32 | 0.0005 |
| 52 | XRCC2 | 3.30 | 0.0006 |
| 53 | SMO | 3.27 | 0.0011 |
| 54 | CDKN3 | 3.26 | 0.0008 |
| 55 | CASC5 | 3.23 | 0.0003 |
| 56 | CBX2 | 3.22 | 0.0006 |
| 57 | TRIP13 | 3.22 | 0.0003 |
| 58 | ANLN | 3.22 | 0.0002 |
| 59 | POLQ | 3.22 | 0.0003 |
| 60 | NOTCH3 | 3.21 | 0.0004 |
| 61 | SOGA2 | 3.21 | 0.0004 |
| 62 | CDC25A | 3.18 | 0.0003 |
| 63 | CEP55 | 3.17 | 0.0001 |
| 64 | UHRF1 | 3.17 | 0.0001 |
| 65 | KIF26A | 3.16 | 0.0006 |
| 66 | MCM10 | 3.12 | 0.0010 |
| 67 | SCN8A | 3.08 | 0.0001 |
| 68 | SLC29A4 | 3.07 | 0.0005 |
| 69 | KIF15 | 3.06 | 0.0002 |
| 70 | SKA3 | 3.06 | 0.0010 |
| 71 | CENPI | 3.05 | 0.0004 |
| 72 | TTK | 3.03 | 0.0005 |
| 73 | NEK2 | 3.03 | 0.0013 |
| 74 | SLCO4A1 | 3.02 | ˂ 0.0001 |
| 75 | CENPE | 3.02 | 0.0005 |
| 76 | CD109 | 2.97 | 0.0001 |
| 77 | CHRNA5 | 2.94 | 0.0008 |
| 78 | KIF18B | 2.91 | 0.0005 |
| 79 | CKAP2L | 2.86 | 0.0015 |
| 80 | CCDC34 | 2.86 | 0.0003 |
| 81 | DLGAP5 | 2.84 | 0.0003 |
| 82 | CDC20 | 2.83 | 0.0004 |
| 83 | BRCA2 | 2.81 | 0.0001 |
| 84 | MELK | 2.79 | 0.0007 |
| 85 | MED12L | 2.79 | 0.0008 |
| 86 | ESCO2 | 2.76 | 0.0008 |
| 87 | MYB | 2.74 | 0.0001 |
| 88 | DTL | 2.73 | 0.0005 |
| 89 | PLK4 | 2.68 | 0.0006 |
| 90 | KIF20A | 2.68 | 0.0012 |
| 91 | MKI67 | 2.66 | 0.0001 |
| 92 | ARHGEF10 | 2.65 | ˂ 0.0001 |
| 93 | ALDH1A2 | 2.62 | 0.0002 |
| 94 | CLSPN | 2.58 | 0.0004 |
| 95 | GINS1 | 2.57 | 0.0003 |
| 96 | MAPT | 2.56 | 0.0002 |
| 97 | TPX2 | 2.53 | 0.0004 |
| 98 | NCAPG | 2.52 | 0.0009 |
| 99 | TYMS | 2.52 | 0.0003 |
| 100 | SCD | 2.51 | 0.0002 |
| 101 | E2F8 | 2.51 | 0.0013 |
| 102 | KIAA1524 | 2.50 | 0.0002 |
| 103 | TTC28 | 2.48 | 0.0008 |
| 104 | VANGL1 | 2.47 | 0.0002 |
| 105 | COX6B2 | 2.47 | 0.0010 |
| 106 | CDC45 | 2.44 | 0.0014 |
| 107 | PLS1 | 2.44 | 0.0007 |
| 108 | HJURP | 2.43 | 0.0009 |
| 109 | TRO | 2.43 | 0.0010 |
| 110 | CTD-2015H6.3 | 2.43 | 0.0006 |
| 111 | LAMP3 | 2.42 | 0.0008 |
| 112 | ZWINT | 2.42 | 0.0003 |
| 113 | ORC6 | 2.39 | 0.0014 |
| 114 | ESPL1 | 2.34 | 0.0007 |
| 115 | ATP8A2 | 2.33 | 0.0004 |
| 116 | IGF2BP3 | 2.33 | 0.0003 |
| 117 | TROAP | 2.33 | 0.0013 |
| 118 | KIF23 | 2.27 | 0.0014 |
| 119 | PSAT1 | 2.26 | 0.0006 |
| 120 | BRIP1 | 2.23 | 0.0005 |
| 121 | CCNA2 | 2.23 | 0.0007 |
| 122 | GUSBP2 | 2.22 | 0.0003 |
| 123 | CCNB1 | 2.22 | 0.0006 |
| 124 | SGOL2 | 2.21 | 0.0013 |
| 125 | FOXM1 | 2.16 | 0.0008 |
| 126 | KIF11 | 2.15 | 0.0010 |
| 127 | TSPYL5 | 2.14 | 0.0014 |
| 128 | UBE2T | 2.12 | 0.0012 |
| 129 | FOXRED2 | 2.08 | 0.0012 |
| 130 | NCAPH | 2.07 | 0.0012 |
| 131 | RRM2 | 2.07 | 0.0009 |
| 132 | NDFIP2 | 2.03 | 0.0005 |
| 133 | CDC42BPA | 1.93 | 0.0006 |
| 134 | CENPU | 1.93 | 0.0015 |
| 135 | AEBP1 | 1.91 | 0.0013 |
| 136 | MAP9 | 1.89 | 0.0012 |
| 137 | BCAT1 | 1.87 | 0.0014 |
| 138 | PRC1 | 1.83 | 0.0007 |
| 139 | SPAG5 | 1.82 | 0.0008 |
| 140 | MTFR2 | 1.76 | 0.0012 |
| 141 | CDK6 | 1.69 | 0.0008 |
| 142 | CELSR3 | 1.67 | 0.0013 |
| 143 | PLK1 | 1.64 | 0.0012 |
| 144 | NUCB2 | 1.63 | 0.0010 |
| 145 | MTHFD1L | 1.57 | 0.0013 |
| 146 | AHI1 | 1.55 | 0.0013 |
| 147 | LAPTM4B | 1.52 | 0.0010 |
| 148 | NCAPG2 | 1.51 | 0.0006 |
| 149 | PAICS | 1.51 | 0.0012 |
| 150 | MTHFD2 | 1.36 | 0.0010 |
| 151 | PCGF6 | 1.34 | 0.0010 |
| 152 | PNPT1 | 1.26 | 0.0013 |
| 153 | DTX1 | 1.07 | 0.0008 |
| 154 | RP1-29C18.8 | 0.92 | 0.0009 |
| 155 | STRBP | 0.88 | 0.0012 |
| 156 | MFHAS1 | 0.62 | 0.0006 |
| 157 | TP53INP2 | -0.73 | 0.0006 |
| 158 | IER3 | -0.74 | 0.0008 |
| 159 | OSER1-AS1 | -0.80 | 0.0004 |
| 160 | RNASE6 | -0.80 | 0.0003 |
| 161 | CLIC3 | -0.90 | 0.0007 |
| 162 | TIGD3 | -0.95 | 0.0001 |
| 163 | GPR162 | -0.96 | 0.0013 |
| 164 | GPX3 | -0.97 | 0.0015 |
| 165 | RCN3 | -1.03 | 0.0003 |
| 166 | MSRB2 | -1.06 | 0.0002 |
| 167 | MAP1LC3A | -1.10 | 0.0009 |
| 168 | KIAA1211L | -1.12 | 0.0007 |
| 169 | TPST1 | -1.32 | ˂ 0.0001 |
| 170 | NECAB2 | -1.38 | 0.0003 |
| 171 | RNA5SP202 | -1.48 | 0.0015 |
| 172 | ALOX15B | -1.53 | 0.0010 |
| 173 | VSIG4 | -1.66 | ˂ 0.0001 |
| 174 | DAAM2 | -1.76 | 0.0013 |
| 175 | IGLV1-44 | -1.89 | 0.0014 |
| 176 | RNU4-2 | -2.13 | 0.0006 |
| 177 | RNY1 | -2.24 | 0.0001 |
| 178 | IGLV3-27 | -2.29 | 0.0002 |
| 179 | IGLV1-47 | -2.75 | ˂ 0.0001 |
| 180 | IGKV1D-16 | -2.77 | 0.0001 |
| 181 | RPL29P11 | -2.92 | 0.0005 |
| 182 | RNY4 | -3.13 | 0.0010 |
| 183 | IGHV6-1 | -3.62 | ˂ 0.0001 |
| 184 | HBZP1 | -3.72 | 0.0002 |
| 185 | AC087650.1 | -4.96 | 0.0004 |
| 186 | PGF | -5.53 | ˂ 0.0001 |
