# Supplementary material for: Integration of Transcriptome and Metabolome Provides Unique Insights to Pathways Associated With Obese Breast Cancer Patients
Source: Front Oncol. 2020 May 19;10:804. doi: 10.3389/fonc.2020.00804 (PMC7248369; doi:10.3389/fonc.2020.00804)
Supplement: Supplementary file 4 [file Table_4.DOCX]

**Supplementary Table S4.** KEGG pathway enrichment of differentially expressed genes in obese compared with non-obese BC patients.

|  | **Term** | **Combined Score** | **Genes** |
| --- | --- | --- | --- |
| 1 | Cell cycle | 209.04 | CDC20; CCNA2; CCNB1; ORC6; CDK6; CDC45; ESPL1; PLK1; CDK1; BUB1B; TTK; CDC25A |
| 2 | Progesterone-mediated oocyte maturation | 52.31 | CCNA2; CCNB1; PLK1; CDK1; ADCY1; CDC25A |
| 3 | One carbon pool by folate | 114.95 | MTHFD1L; MTHFD2; TYMS |
| 4 | Oocyte meiosis | 35.06 | CDC20; CCNB1; ESPL1; PLK1; CDK1; ADCY1 |
| 5 | Cellular senescence | 22.38 | CCNA2; CCNB1; CDK6; CDK1; FOXM1; CDC25A |
| 6 | p53 signaling pathway | 32.21 | CCNB1; RRM2; CDK6; CDK1 |
| 7 | Homologous recombination | 39.61 | BRIP1; XRCC2; BRCA2 |
| 8 | Fanconi anemia pathway | 25.56 | BRIP1; UBE2T; BRCA2 |
| 9 | Human T-cell leukemia virus 1 infection | 7.16 | CDC20; CCNA2; ESPL1; BUB1B; ADCY1 |
| 10 | Vitamin B6 metabolism | 52.13 | PSAT1 |
| 11 | Notch signaling pathway | 11.70 | NOTCH3; DTX1 |
| 12 | Glutathione metabolism | 9.01 | RRM2; GPX3 |
| 13 | Pyrimidine metabolism | 8.74 | RRM2; TYMS |
| 14 | Arachidonic acid metabolism | 7.34 | GPX3; ALOX15B |
| 15 | Viral carcinogenesis | 4.57 | CDC20; CCNA2; CDK6; CDK1 |
| 16 | Purine metabolism | 5.32 | RRM2; ADCY1; PAICS |
| 17 | FoxO signaling pathway | 5.08 | PLK4; CCNB1; PLK1 |
| 18 | Apelin signaling pathway | 4.70 | NOTCH3; ADCY1; SLC8A2 |
| 19 | Thyroid hormone synthesis | 5.49 | GPX3; ADCY1 |
| 20 | Pancreatic cancer | 5.36 | CDK6; BRCA2 |
| 21 | Breast cancer | 4.06 | NOTCH3; CDK6; BRCA2 |
| 22 | Pantothenate and CoA biosynthesis | 10.28 | BCAT1 |
| 23 | Gap junction | 3.97 | CDK1; ADCY1 |
| 24 | Protein digestion and absorption | 3.80 | COL27A1; SLC8A2 |
| 25 | Biosynthesis of unsaturated fatty acids | 5.97 | SCD |
| 26 | Axon guidance | 2.56 | ENAH; SMO; DPYSL5 |
| 27 | Mucin type O-glycan biosynthesis | 4.79 | GALNT9 |
| 28 | Cholinergic synapse | 2.45 | CHRNA3; ADCY1 |
| 29 | Thyroid hormone signaling pathway | 2.27 | NOTCH3; MED12L |
| 30 | MicroRNAs in cancer | 1.72 | NOTCH3; CDK6; KIF23; CDC25A |
| 31 | AMPK signaling pathway | 2.12 | CCNA2; SCD |
| 32 | Ferroptosis | 3.13 | MAP1LC3A |
| 33 | Glycine, serine and threonine metabolism | 3.13 | PSAT1 |
| 34 | Lysosome | 2.01 | LAPTM4B; LAMP3 |
| 35 | Cysteine and methionine metabolism | 2.36 | BCAT1 |
| 36 | Hedgehog signaling pathway | 2.36 | SMO |
| 37 | Valine, leucine and isoleucine degradation | 2.28 | BCAT1 |
| 38 | Thermogenesis | 1.41 | DPF1; ADCY1; COX6B2 |
| 39 | Ovarian steroidogenesis | 2.20 | ADCY1 |
| 40 | Pathways in cancer | 1.21 | NOTCH3; CDK6;SMO; ADCY1; BRCA2; PGF |
| 41 | Glycosaminoglycan biosynthesis | 1.91 | NDST3 |
| 42 | Regulation of lipolysis in adipocytes | 1.78 | ADCY1 |
| 43 | Cushing syndrome | 1.19 | CDK6; ADCY1 |
| 44 | Wnt signaling pathway | 1.14 | VANGL1; DAAM2 |
| 45 | Basal cell carcinoma | 1.38 | SMO |
| 46 | Cortisol synthesis and secretion | 1.30 | ADCY1 |
| 47 | cGMP-PKG signaling pathway | 1.01 | ADCY1; SLC8A2 |
| 48 | Non-small cell lung cancer | 1.26 | CDK6 |
| 49 | Hepatocellular carcinoma | 0.98 | CDK6; DPF1 |
| 50 | Long-term potentiation | 1.23 | ADCY1 |
| 51 | Retinol metabolism | 1.23 | ALDH1A2 |
| 52 | Alzheimer disease | 0.94 | MAPT; COX6B2 |
| 53 | Bile secretion | 1.06 | ADCY1 |
| 54 | Melanoma | 1.06 | CDK6 |
| 55 | Bacterial invasion of epithelial cells | 1.01 | SEPT3 |
| 56 | PPAR signaling pathway | 1.01 | SCD |
| 57 | Gastric acid secretion | 0.98 | ADCY1 |
| 58 | Glioma | 0.98 | CDK6 |
| 59 | Chronic myeloid leukemia | 0.96 | CDK6 |
| 60 | Cardiac muscle contraction | 0.91 | COX6B2 |
| 61 | Complement and coagulation cascades | 0.88 | VSIG4 |
| 62 | RNA degradation | 0.88 | PNPT1 |
| 63 | Calcium signaling pathway | 0.74 | ADCY1; SLC8A2 |
| 64 | ECM-receptor interaction | 0.82 | HMMR |
| 65 | Peroxisome | 0.80 | PEX5L |
| 66 | Insulin secretion | 0.74 | ADCY1 |
| 67 | Salmonella infection | 0.74 | MYH10 |
| 68 | Epstein-Barr virus infection | 0.62 | CCNA2; CDK6 |
| 69 | GABAergic synapse | 0.69 | ADCY1 |
| 70 | Salivary secretion | 0.67 | ADCY1 |
| 71 | Rap1 signaling pathway | 0.58 | ADCY1; PGF |
| 72 | Dilated cardiomyopathy (DCM) | 0.66 | ADCY1 |
| 73 | Morphine addiction | 0.66 | ADCY1 |
| 74 | Th1 and Th2 cell differentiation | 0.64 | NOTCH3 |
| 75 | GnRH signaling pathway | 0.63 | ADCY1 |
| 76 | Small cell lung cancer | 0.63 | CDK6 |
| 77 | Human immunodeficiency virus 1 infection | 0.54 | CCNB1;CDK1 |
| 78 | Amoebiasis | 0.59 | ADCY1 |
| 79 | Regulation of actin cytoskeleton | 0.52 | ENAH; MYH10 |
| 80 | Human papillomavirus infection | 0.51 | CCNA2; NOTCH3; CDK6 |
| 81 | Circadian entrainment | 0.57 | ADCY1 |
| 82 | Aldosterone synthesis and secretion | 0.56 | ADCY1 |
| 83 | Pancreatic secretion | 0.56 | ADCY1 |
| 84 | Inflammatory mediator regulation of TRP channels | 0.53 | ADCY1 |
| 85 | Melanogenesis | 0.52 | ADCY1 |
| 86 | Longevity regulating pathway | 0.51 | ADCY1 |
| 87 | Chagas disease (American trypanosomiasis) | 0.50 | ADCY1 |
| 88 | Human cytomegalovirus infection | 0.45 | CDK6; ADCY1 |
| 89 | Parathyroid hormone synthesis, secretion and action | 0.47 | ADCY1 |
| 90 | Drug metabolism | 0.45 | RRM2 |
| 91 | Ras signaling pathway | 0.42 | RASAL1; PGF |
| 92 | PI3K-Akt signaling pathway | 0.40 | CDK6; MYB; PGF |
| 93 | Leukocyte transendothelial migration | 0.41 | THY1 |
| 94 | Serotonergic synapse | 0.41 | ALOX15B |
| 95 | Glutamatergic synapse | 0.40 | ADCY1 |
| 96 | Platelet activation | 0.33 | ADCY1 |
| 97 | Autophagy | 0.30 | TP53INP2 |
| 98 | Relaxin signaling pathway | 0.29 | ADCY1 |
| 99 | Vascular smooth muscle contraction | 0.28 | ADCY1 |
| 100 | Oxidative phosphorylation | 0.27 | COX6B2 |
| 101 | Estrogen signaling pathway | 0.25 | ADCY1 |
| 102 | Ubiquitin mediated proteolysis | 0.25 | CDC20 |
| 103 | Measles | 0.25 | CDK6 |
| 104 | Signaling pathways regulating pluripotency of stem cells | 0.25 | PCGF6 |
| 105 | Parkinson disease | 0.23 | COX6B2 |
| 106 | Adrenergic signaling in cardiomyocytes | 0.22 | ADCY1 |
| 107 | Phospholipase D signaling pathway | 0.21 | ADCY1 |
| 108 | Retrograde endocannabinoid signaling | 0.21 | ADCY1 |
| 109 | Non-alcoholic fatty liver disease (NAFLD) | 0.20 | COX6B2 |
| 110 | Phagosome | 0.19 | COLEC11 |
| 111 | Oxytocin signaling pathway | 0.19 | ADCY1 |
| 112 | MAPK signaling pathway | 0.20 | MAPT; PGF |
| 113 | Hepatitis C | 0.18 | CDK6 |
| 114 | Hepatitis B | 0.16 | CCNA2 |
| 115 | Protein processing in endoplasmic reticulum | 0.16 | HSPA4L |
| 116 | Tight junction | 0.14 | MYH10 |
| 117 | Neuroactive ligand-receptor interaction | 0.12 | CHRNA3; CHRNA5 |
| 118 | Kaposi sarcoma-associated herpesvirus infection | 0.11 | CDK6 |
| 119 | Transcriptional misregulation in cancer | 0.11 | TSPAN7 |
| 120 | Chemokine signaling pathway | 0.10 | ADCY1 |
| 121 | Huntington disease | 0.10 | COX6B2 |
| 122 | Focal adhesion | 0.09 | PGF |
| 123 | Proteoglycans in cancer | 0.09 | SMO |
| 124 | cAMP signaling pathway | 0.07 | ADCY1 |
| 125 | Olfactory transduction | 0.01 | SLC8A2 |
