# Supplementary material for: Integration of Transcriptome and Metabolome Provides Unique Insights to Pathways Associated With Obese Breast Cancer Patients
Source: Front Oncol. 2020 May 19;10:804. doi: 10.3389/fonc.2020.00804 (PMC7248369; doi:10.3389/fonc.2020.00804)
Supplement: Supplementary file 5 [file Table_5.DOCX]

**Supplementary Table S5.** The detailed lists of significantly deregulated metabolites in obese compared with non-obese BC patients, ordered depending on FC. FC rounded 2 numbers after the decimal point. FC: Fold change.

|  | **Metabolite** | **FC** | ***p*-value** | **Regulation** |
| --- | --- | --- | --- | --- |
| 1 | SM(d19:1/24:1(15Z)) | 93.56 | 0.0008 | Up |
| 2 | (8E)-Dotriacont-8-enoic acid | 20.99 | ˂ 0.0001 | Up |
| 3 | gamma-Hydroxyarginine | 10.61 | ˂ 0.0001 | Up |
| 4 | Guanidine | 7.80 | 0.0005 | Up |
| 5 | 12-Tricosanol | 7.33 | ˂ 0.0001 | Up |
| 6 | {2,6-dihydroxy-4-[({6,7,8,11,12,13,22,23-octahydroxy-3,16-dioxo-2,17,20-trioxatetracyclo[17.3.1.0â´,â¹.0Â¹â°,Â¹âµ]tricosa-4,6,8,10,12,14-hexaen-21-yl}oxy)carbonyl]phenyl}oxidanesulfonic acid | 6.22 | 0.0001 | Up |
| 7 | DL-Cerebronic acid | 5.99 | ˂ 0.0001 | Up |
| 8 | Thromboxane | 5.44 | ˂ 0.0001 | Up |
| 9 | Ornithine | 5.36 | ˂ 0.0001 | Up |
| 10 | 7-Methyladenine | 5.27 | ˂ 0.0001 | Up |
| 11 | Glycerol 3-phosphate | 5.21 | ˂ 0.0001 | Up |
| 12 | D-Leucine | 5.06 | ˂ 0.0001 | Up |
| 13 | 25-Hydroxycholesterol | 4.96 | ˂ 0.0001 | Up |
| 14 | Paclitaxel | 4.03 | ˂ 0.0001 | Up |
| 15 | Juniperic acid | 4.00 | ˂ 0.0001 | Up |
| 16 | 3-Eicosyne | 3.56 | ˂ 0.0001 | Up |
| 17 | L-Carnitine | 3.55 | ˂ 0.0001 | Up |
| 18 | Piperidine | 3.46 | ˂ 0.0001 | Up |
| 19 | 2-Oxoarginine | 3.34 | ˂ 0.0001 | Up |
| 20 | N-Acetylhistamine | 2.98 | 0.0031 | Up |
| 21 | Pentadecane | 2.80 | 0.0298 | Up |
| 22 | Thymine | 2.37 | 0.0004 | Up |
| 23 | Sulcatone | 2.29 | 0.0001 | Up |
| 24 | Nervonic acid | 2.22 | 0.0005 | Up |
| 25 | Sapiol | 2.18 | 0.0005 | Up |
| 26 | Dihydrouracil | 1.87 | 0.0086 | Up |
| 27 | Oleamide | 1.79 | 0.0029 | Up |
| 28 | Arachidic acid | 1.78 | 0.0036 | Up |
| 29 | Methylguanidine | 1.73 | 0.0104 | Up |
| 30 | Histamine | 1.69 | 0.0026 | Up |
| 31 | Serotonin | 1.66 | 0.0003 | Up |
| 32 | L-2-Hydroxyglutaric acid | 1.64 | 0.0004 | Up |
| 33 | Acetylcholine | 1.46 | 0.0068 | Up |
| 34 | 8-Methylnonenoate | 1.37 | 0.0001 | Up |
| 35 | L-Homoserine | 1.35 | 0.0375 | Up |
| 36 | Tryptophan | 1.34 | 0.0236 | Up |
| 37 | Coenzyme Q9 | 233.78 | ˂ 0.0001 | Down |
| 38 | Cholestenone | 230.43 | ˂ 0.0001 | Down |
| 39 | Nonacosanol | 111.33 | ˂ 0.0001 | Down |
| 40 | 10Z-Nonadecenoic acid | 82.10 | ˂ 0.0001 | Down |
| 41 | Palmitaldehyde | 68.94 | ˂ 0.0001 | Down |
| 42 | Dodecane | 65.96 | ˂ 0.0001 | Down |
| 43 | Delta-Tocopherol | 47.27 | ˂ 0.0001 | Down |
| 44 | Myristoleic acid | 32.33 | ˂ 0.0001 | Down |
| 45 | 3-Hydroxyphenylacetic acid | 31.27 | ˂ 0.0001 | Down |
| 46 | 2-Undecanol | 27.21 | ˂ 0.0001 | Down |
| 47 | L-Leucine | 14.32 | ˂ 0.0001 | Down |
| 48 | Decamethonium | 8.72 | 0.0055 | Down |
| 49 | Erucoylacetone | 6.55 | ˂ 0.0001 | Down |
| 50 | Geddyl alcohol | 5.65 | ˂ 0.0001 | Down |
| 51 | 4-Methylnonacosane | 5.22 | 0.0001 | Down |
| 52 | Guanosine triphosphate adenosine | 4.97 | ˂ 0.0001 | Down |
| 53 | FAD | 4.87 | 0.0002 | Down |
| 54 | Hexadecenal | 3.90 | 0.0538 | Down |
| 55 | Pentanoyl-CoA | 3.86 | 0.0001 | Down |
| 56 | Monoethanolamine oleate | 3.76 | ˂ 0.0001 | Down |
| 57 | 24-Propylcholestan-3-ol | 3.73 | 0.0011 | Down |
| 58 | Hexanoyl-CoA | 3.28 | 0.0004 | Down |
| 59 | 8-Dotriacontenoic acid | 3.08 | ˂ 0.0001 | Down |
| 60 | Palmitone | 2.93 | 0.0018 | Down |
| 61 | TG(16:0/14:1(9Z)/18:4(6Z,9Z,12Z,15Z)) | 2.87 | 0.0006 | Down |
| 62 | TG(20:0/o-18:0/20:0) | 2.62 | 0.0026 | Down |
| 63 | Carbamoyl phosphate | 2.56 | 0.0001 | Down |
| 64 | erythro-8,10-Tritriacontanediol | 2.56 | 0.0007 | Down |
| 65 | 1-Hexacosene | 2.46 | ˂ 0.0001 | Down |
| 66 | Nonadecanol | 2.30 | 0.0354 | Down |
| 67 | Hentriacontane | 2.17 | 0.0001 | Down |
| 68 | N1-Acetylspermine | 2.17 | 0.0409 | Down |
| 69 | Chlordecone alcohol | 2.05 | 0.0007 | Down |
| 70 | Nonacosane | 2.03 | 0.0006 | Down |
| 71 | Cer(d18:0/18:0) | 2.03 | 0.0166 | Down |
| 72 | CL(16:1(9Z)/18:1(9Z)/16:1(9Z)/16:1(9Z)) | 1.89 | 0.0025 | Down |
| 73 | Undecanoylcholine | 1.82 | 0.0178 | Down |
| 74 | Cetyl alcohol | 1.78 | 0.0001 | Down |
| 75 | erythro-8,10-Heptacosanediol | 1.77 | ˂ 0.0001 | Down |
| 76 | Lactosylcermide (d18:1/20:0) | 1.76 | 0.0325 | Down |
| 77 | 7a-Hydroxy-cholestene-3-one | 1.75 | 0.0063 | Down |
| 78 | Alpha-Linolenic acid | 1.71 | 0.0019 | Down |
| 79 | Coenzyme m | 1.70 | 0.0027 | Down |
| 80 | 1-Dodecene | 1.67 | ˂ 0.0001 | Down |
| 81 | 14-Heptacosanol | 1.64 | 0.0011 | Down |
| 82 | jasmonic acid | 1.62 | ˂ 0.0001 | Down |
| 83 | Heptadecane | 1.51 | 0.0255 | Down |
| 84 | Homovanillic acid | 1.40 | ˂ 0.0001 | Down |
| 85 | L-Palmitoylcarnitine | 1.39 | 0.0038 | Down |
| 86 | Progesterone | 1.37 | 0.0064 | Down |
| 87 | 5,10-Pentadecadien-1-ol | 1.35 | ˂ 0.0001 | Down |
| 88 | N-Acetyl-L-tyrosine | 1.34 | 0.0172 | Down |
| 89 | gamma-Terpineol | 1.34 | 0.0026 | Down |
| 90 | Creatinine | 1.32 | 0.0110 | Down |
| 91 | 2,6,10,14-Tetramethylpentadecan-6-ol | 1.31 | 0.0378 | Down |
| 92 | PI(16:0/18:0) | 1.31 | 0.0063 | Down |
| 93 | Epinephrine | 1.25 | 0.0003 | Down |
| 94 | Pentadecanal | 1.25 | 0.0055 | Down |
| 95 | Schottenol | 1.24 | 0.0437 | Down |
| 96 | 7-Dehydrodesmosterol | 1.19 | 0.0445 | Down |
