# Supplementary material for: Integration of Transcriptome and Metabolome Provides Unique Insights to Pathways Associated With Obese Breast Cancer Patients
Source: Front Oncol. 2020 May 19;10:804. doi: 10.3389/fonc.2020.00804 (PMC7248369; doi:10.3389/fonc.2020.00804)
Supplement: Supplementary file 6 [file Table_6.DOCX]

**Supplementary Table S6.** Pathway enrichment of differential metabolites in obese compared with non-obese BC patents.

|  | **Pathways** | **Total** | **Expected** | **Hits** |
| --- | --- | --- | --- | --- |
| 1 | D-Arginine and D-Ornithine Metabolism | 11 | 0.83 | 2 |
| 2 | Glycerol Phosphate Shuttle | 11 | 0.83 | 2 |
| 3 | Mitochondrial Beta-Oxidation of Short Chain Saturated Fatty Acids | 27 | 2.03 | 3 |
| 4 | Phospholipid Biosynthesis | 29 | 2.18 | 3 |
| 5 | Fatty acid Metabolism | 43 | 3.23 | 4 |
| 6 | Mitochondrial Electron Transport Chain | 19 | 1.43 | 2 |
| 7 | De Novo Triacylglycerol Biosynthesis | 9 | 0.68 | 1 |
| 8 | Pyruvaldehyde Degradation | 10 | 0.75 | 1 |
| 9 | Glycerolipid Metabolism | 25 | 1.88 | 2 |
| 10 | Cardiolipin Biosynthesis | 11 | 0.83 | 1 |
| 11 | Plasmalogen Synthesis | 26 | 1.95 | 2 |
| 12 | Mitochondrial Beta-Oxidation of Medium Chain Saturated Fatty Acids | 27 | 2.03 | 2 |
| 13 | Mitochondrial Beta-Oxidation of Long Chain Saturated Fatty Acids | 28 | 2.1 | 2 |
| 14 | Thyroid hormone synthesis | 13 | 0.98 | 1 |
| 15 | Urea Cycle | 29 | 2.18 | 2 |
| 16 | Pyrimidine Metabolism | 59 | 4.43 | 4 |
| 17 | Vitamin K Metabolism | 14 | 1.05 | 1 |
| 18 | Ammonia Recycling | 32 | 2.4 | 2 |
| 19 | Beta Oxidation of Very Long Chain Fatty Acids | 17 | 1.28 | 1 |
| 20 | Beta-Alanine Metabolism | 34 | 2.55 | 2 |
| 21 | Aspartate Metabolism | 35 | 2.63 | 2 |
| 22 | Spermidine and Spermine Biosynthesis | 18 | 1.35 | 1 |
| 23 | Alpha Linolenic Acid and Linoleic Acid Metabolism | 19 | 1.43 | 1 |
| 24 | Butyrate Metabolism | 19 | 1.43 | 1 |
| 25 | Arginine and Proline Metabolism | 53 | 3.98 | 3 |
| 26 | Catecholamine Biosynthesis | 20 | 1.5 | 1 |
| 27 | Riboflavin Metabolism | 20 | 1.5 | 1 |
| 28 | Threonine and 2-Oxobutanoate Degradation | 20 | 1.5 | 1 |
| 29 | Vitamin B6 Metabolism | 20 | 1.5 | 1 |
| 30 | Betaine Metabolism | 21 | 1.58 | 1 |
| 31 | Glutathione Metabolism | 21 | 1.58 | 1 |
| 32 | Carnitine Synthesis | 22 | 1.65 | 1 |
| 33 | Tryptophan Metabolism | 60 | 4.51 | 3 |
| 34 | Caffeine Metabolism | 24 | 1.8 | 1 |
| 35 | Histidine Metabolism | 43 | 3.23 | 2 |
| 36 | Methionine Metabolism | 43 | 3.23 | 2 |
| 37 | Oxidation of Branched Chain Fatty Acids | 26 | 1.95 | 1 |
| 38 | Bile Acid Biosynthesis | 65 | 4.88 | 3 |
| 39 | Steroid Biosynthesis | 48 | 3.61 | 2 |
| 40 | Phenylalanine and Tyrosine Metabolism | 28 | 2.1 | 1 |
| 41 | Glutamate Metabolism | 49 | 3.68 | 2 |
| 42 | Folate Metabolism | 29 | 2.18 | 1 |
| 43 | Lysine Degradation | 30 | 2.25 | 1 |
| 44 | Citric Acid Cycle | 32 | 2.4 | 1 |
| 45 | Tyrosine Metabolism | 72 | 5.41 | 3 |
| 46 | Fatty Acid Elongation In Mitochondria | 35 | 2.63 | 1 |
| 47 | Glycine and Serine Metabolism | 59 | 4.43 | 3 |
| 48 | Nicotinate and Nicotinamide Metabolism | 37 | 2.78 | 1 |
| 49 | Valine, Leucine and Isoleucine Degradation | 60 | 4.51 | 2 |
| 50 | Porphyrin Metabolism | 40 | 3 | 1 |
| 51 | Sphingolipid Metabolism | 40 | 3 | 1 |
| 52 | Propanoate Metabolism | 42 | 3.16 | 1 |
| 53 | Steroidogenesis | 43 | 3.23 | 1 |
| 54 | Pyruvate Metabolism | 48 | 3.61 | 1 |
| 55 | Warburg Effect | 58 | 4.36 | 1 |
| 56 | Purine Metabolism | 74 | 5.56 | 1 |
