# Supplementary material for: Integration of Transcriptome and Metabolome Provides Unique Insights to Pathways Associated With Obese Breast Cancer Patients
Source: Front Oncol. 2020 May 19;10:804. doi: 10.3389/fonc.2020.00804 (PMC7248369; doi:10.3389/fonc.2020.00804)
Supplement: Supplementary file 7 [file Table_7.DOCX]

**Supplementary Table S7.** Differentially expressed genes and identified altered metabolites that enriched in overlap integrated pathways. FC: fold change

|  | **Pathways altered** | **Transcripts enriched** | | | | **Metabolites enriched** | | |
| --- | --- | --- | --- | --- | --- | --- | --- | --- |
|  |  | **Gene symbol** | **Gene name** | **LogFC** | ***p*-value** | **Metabolite** | **LogFC** | ***p*-value** |
| 1 | Glutathione metabolism | RRM2 | Ribonucleotide Reductase Regulatory Subunit M2 | 2.07 | 0.0009 | FAD | -2.28 | 0.0002 |
|  |  | GPX3 | Glutathione peroxidase 3 | -0.97 | 0.002 |  |  |  |
| 2 | Glycine and Serine Metabolism | PSAT1 | Phosphoserine Aminotransferase 1 | 2.26 | 0.0006 | Ornithine | 2.42 | ˂0.0001 |
|  |  |  |  |  |  | FAD | -2.28 | 0.0002 |
|  |  |  |  |  |  | L-Leucine | -3.84 | ˂0.0001 |
| 3 | Purine metabolism | RRM2 | Ribonucleotide Reductase Regulatory Subunit M2 | 2.07 | 0.0009 | FAD | -2.28 | 0.0002 |
|  |  | ADCY1 | adenylate cyclase 1 | 5.52 | 0.0001 |  |  |  |
|  |  | PAICS | phosphoribosylaminoimidazole carboxylase and phosphoribosylaminoimidazolesuccinocarboxamide synthase | 1.51 | 0.001 |  |  |  |
| 4 | Pyrimidine metabolism | RRM2 | Ribonucleotide Reductase Regulatory Subunit M2 | 2.07 | 0.0009 | Dihydrouracil | 0.90 | 0.009 |
|  |  |  |  |  |  | Thymine | 1.24 | 0.0004 |
|  |  | TYMS | Thymidylate Synthetase | 2.52 | 0.0003 | Carbamoyl phosphate | -1.36 | 0.0001 |
|  |  |  |  |  |  | FAD | -2.28 | 0.0002 |
| 5 | Thyroid hormone synthesis | GPX3 | Glutathione Peroxidase 3 | -0.97 | 0.002 | FAD | -2.28 | 0.0002 |
|  |  | ADCY1 | adenylate cyclase 1 | 5.52 | 0.0001 |  |  |  |
| 6 | Valine, Leucine and Isoleucine degradation | BCAT1 | Branched Chain Amino Acid Transaminase 1 | 1.87 | 0.0014 | L-Leucine | -3.84 | ˂0.0001 |
|  |  |  |  |  |  | FAD | -2.28 | 0.0002 |
| 7 | Vitamin B6 metabolism | PSAT1 | Phosphoserine Aminotransferase 1 | 2.26 | 0.0006 | FAD | -2.28 | 0.0002 |
